# Design, synthesis and antiproliferative activity of pyridone derivatives as potent enhancer of zeste homologue 2/histone deacetylase dual inhibitors

**DOI:** 10.1039/d6ra04928a

**Published:** 2026-08-11

**Authors:** Zhaobang Tan, Jiajun Ding, Xin Chen, Lei Qian, Qiang Yu

**Affiliations:** a Department of Digestive Surgery, Xijing Hospital of Digestive Diseases, Fourth Military Medical University Xi'an 710032 China yq1744585238@163.com +86-29-84775507; b Department of Thyroid, Breast and Vascular Surgery, Xijing Hospital, Fourth Military Medical University Xi'an 710032 China; c Shaanxi Key Laboratory of Natural Products & Chemical Biology, College of Chemistry & Pharmacy, Northwest A&F University Yangling 712100 P.R. China chenxin1888@nwsuaf.edu.cn +86-29-87092335; d Department of Experimental Surgery, Xijing Hospital, Fourth Military Medical University Xi'an 710032 China rayqian0307@foxmail.com; e College of Food Science and Engineering, Northwest A&F University Yangling 712100 China

## Abstract

The development of dual inhibitors of histone deacetylases (HDACs) and enhancer of zeste homologue 2 (EZH2) is an efficient strategy that not only synergistically suppresses critical pathways in tumorigenesis but also circumvents the potential risks of drug cocktails. In this study, a series of pyridone derivatives were rationally designed *via* pharmacophore merging, and *N*^1^-((4,6-dimethyl-2-oxo-1,2-dihydropyridin-3-yl)methyl)-*N*^8^-hydroxyoctanediamide (15c) was identified as the most potent compound against hematological tumor cells MV4-11 and SU-DHL-10, with IC_50_ values in the submicromolar range. 15c also effectively inhibited HDAC1 and EZH2 with IC_50_ values of 9.2 nM and 311.1 nM, respectively. Molecular simulations revealed key interactions between 15c and both targets. These findings indicated that compound 15c warrants further investigation as a novel dual HDAC/EZH2 agent.

## Abstract 2

The pyridone-based compound 15c emerged as a dual HDAC/EZH2 inhibitor, exhibiting submicromolar antiproliferative activity and potent enzymatic inhibition, with molecular simulations rationalizing its binding mode to both targets.

## Graphical abstract

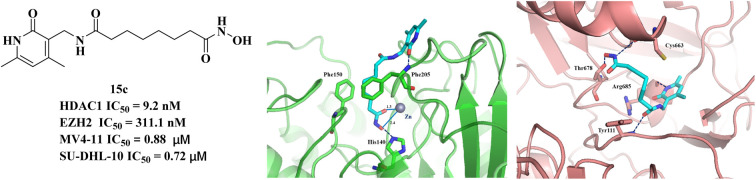

## 1 Introduction

**Fig. 1:**
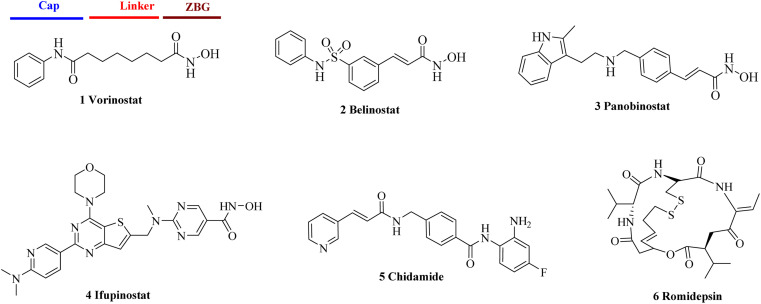
Approved HDAC inhibitors.

Histone tail modifications constitute a major mechanism of epigenetic regulation, governing the switch between gene activation and silencing.^1^ Among these processes, acetylation and methylation occurring at the lysine-rich *N*-terminal tails of histone proteins play a dominant role in modulating chromatin structure and subsequent gene transcription.^2–4^ Histone deacetylases (HDACs), which regulate the acetylation of histones and cytoplasmic proteins, are found to be overexpressed in many types of cancer.^5^ Inhibition of HDACs exerts anti-cancer effects through multiple mechanisms including reduced cell motility/migration, suppressed invasion, induced apoptosis, inhibited angiogenesis, and blocked DNA repair.^6^ The eleven classical zinc-dependent HDACs fall into four distinct subclasses: class I (HDAC1/2/3/8), class IIa (HDAC4/5/7/9), class IIb (HDAC6/10), and class IV (HDAC11).^7,8^ Notably, class I HDACs are widespread across all tissues; hence, most HDAC inhibitors (HDACis) in clinical use target class I HDACs.^9–11^ HDACis share a well-established pharmacophore comprising three components: a capping motif outside the active pocket, a zinc-binding group (ZBG) chelating the catalytically active zinc ion at the bottom of the pocket, and a linker that bridges the cap and ZBG (Fig. 1).^12–15^ Hydroxamic acid is a common nonselective ZBG, and four prominent hydroxamic acid-based compounds have been approved for cancer treatment: vorinostat (1),^16^ belinostat (2),^17^ panobinostat (3)^18^ and ifupinostat (4). *O*-phenylenediamine and its derivatives represent another important class of ZBGs, which display distinct selectivity toward class I HDACs.^19,20^ For example, chidamide (5) was approved for treating recurrent and refractory peripheral T-cell lymphoma by the NMPA in 2015.^21,22^ Natural macrocyclic romidepsin (6) features a unique disulfide as its ZBG and has gained FDA approval for the treatment of cutaneous or peripheral T-cell lymphoma and multiple myeloma.^23^

**Fig. 2:**
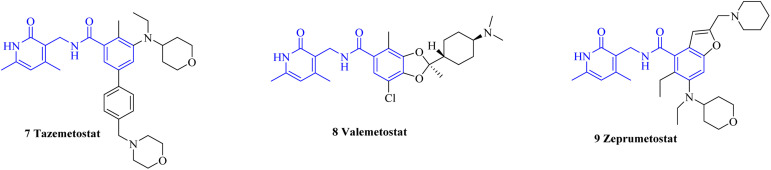
Approved EZH2 inhibitors.

Enhancer of zeste homolog 2 (EZH2) is another key epigenetic enzyme.^24,25^ As the catalytic subunit of polycomb repressive complex 2 (PRC2), it primarily mediates histone H3 lysine 27 (H3K27) methylation. EZH2 has been found to be overexpressed or mutated in a variety of solid tumors and hematological malignancies and is closely related to cell proliferation, cell-cycle, cell differentiation, and self-renewal.^26,27^ Thus far, a number of EZH2 inhibitors have been described,^28^ three of which are available in the market (Fig. 2). Tazemetostat (7), a first-in-class EZH2 inhibitor,^29^ was approved in 2020 by the FDA for the treatment of relapsed/refractory follicular lymphoma.^30^ However, it was recently withdrawn due to the risk of secondary hematologic malignancies. Valemetostat (8) is an orally bioavailable EZH1/2 dual inhibitor that was approved in Japan in 2022 for the treatment of relapsed or refractory T-cell leukemia/lymphoma.^31^ It inhibits EZH1 and EZH2 with IC_50_ values of 8.4 and 2.5 nM, respectively, and shows antiproliferative activity against KARPAS-422 cells with a GI_50_ value of 4.8 nM.^32^ Zeprumetostat (9) was approved for use in adult patients with relapsed or refractory peripheral T-cell lymphoma in 2025.^33,34^ It is a potent and highly selective EZH2 inhibitor that specifically inhibits both wild-type EZH2 and mutant EZH2^Y641F^ with IC_50_ values of 0.87 nM and 2.68 nM, respectively. It can induce G1 phase arrest and promote apoptosis in a diffuse large B-cell lymphoma (DLBCL) cell line with IC_50_ values of <300 nM.

**Fig. 3:**
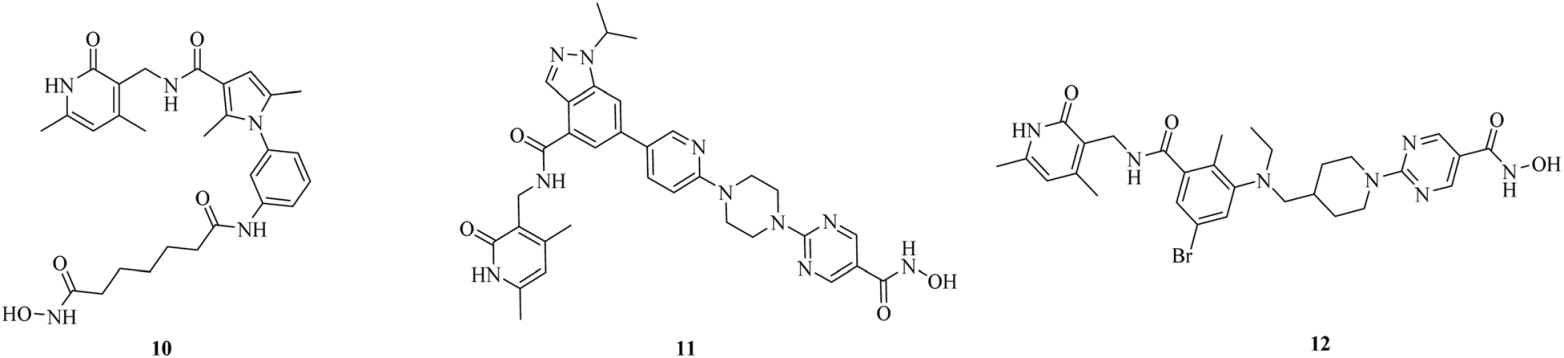
Reported EZH2/HDAC dual inhibitors.

The dual inhibition of EZH2 and HDACs has been reported to exert combined antiproliferative effects against hematological malignancies by simultaneously regulating the acetylation and methylation of H3K27. Zeprumetostat enhanced the antitumor efficacy of chidamide through STAT1 in T-cell lymphoma^35^ and DLBCL.^36^ The combination of romidepsin and an EZH2 inhibitor also demonstrated potent synergy in lymphoma cell line.^37^ Treatment with the EZH2 inhibitor 3-deazaneplanocin A and panobinostat synergistically induced apoptosis in cultured and primary acute myeloid leukemia (AML) cells.^38^ Epigenetic pathways have been implicated in mediating castration-resistant prostate cancer (CRPC). A new study has shown that the dual inhibition of EZH2 and HDACs may sensitize CRPC to both epigenetic and standard therapies.^39^ These findings support combined HDAC/EZH2 inhibition as a promising epigenetic therapeutic strategy for treating hematological malignancies. Recent reports suggest that fusing the pharmacophores of HDAC and EZH2 inhibitors into a single molecule is feasible (Fig. 3). Annalisa *et al.* reported a first-in-class dual EZH2/HDAC inhibitor (10), which reduced leukemia and rhabdomyosarcoma cell viability at low micromolar concentrations.^40^ Compound 11 potently inhibited HDAC1 (IC_50_ = 0.12 µM) and EZH2 (IC_50_ = 0.059 µM) and exhibited good antiproliferative activity against leukemia cells MV4-11 (IC_50_ = 0.17 µM).^41^ Zhang *et al.* also disclosed a tazemetostat-based HDAC/EZH2 dual inhibitor (12), which was effective against lymphoma cell lines and multiple AML cell lines.^42^

## 2 Compound design

**Fig. 4:**
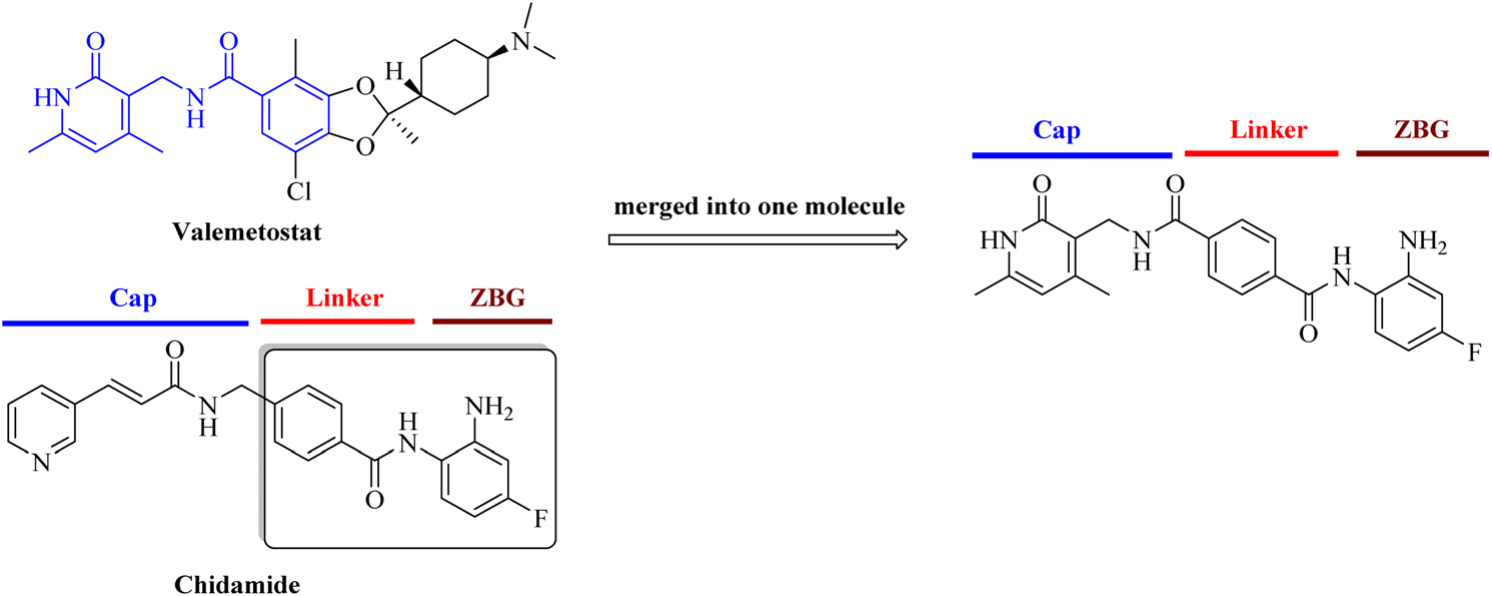
Design of pyridone-based EZH2/HDAC dual inhibitors.

Common strategies for multi-target drug design include developing conjugated, fused, or merged pharmacophores.^43,44^ The structural flexibility of HDACis makes them easy to hybridize with other agents.^10,45–50^ However, the reported dual HDAC/EZH2 inhibitors were all obtained *via* pharmacophore fusion, and such inhibitors generally have high molecular weights, resulting in poor absorption and low oral bioavailability.^51,52^ Hence, a hybrid molecule with a lower molecular weight obtained by merging pharmacophores has distinct advantages. Structural analysis of EZH2 inhibitors 7–9 revealed that 2-pyridone was a key fragment. In addition, they shared a common benzamide scaffold, and the phenyl could also be used as the linker part of chidamide (Fig. 4). Hence, a novel dual inhibitor was developed by replacing only the pyridine-alkene capping group of chidamide with 2-pyridone while retaining the phenyl linker and *o*-phenylenediamine ZBG. Moreover, a classical hydroxamic acid group and an aliphatic chain linker were employed. Here, we reported the synthesis, structure–activity relationship (SAR) study and antiproliferative evaluation of these new pyridones.

## 3 Chemistry

**Scheme 1:**
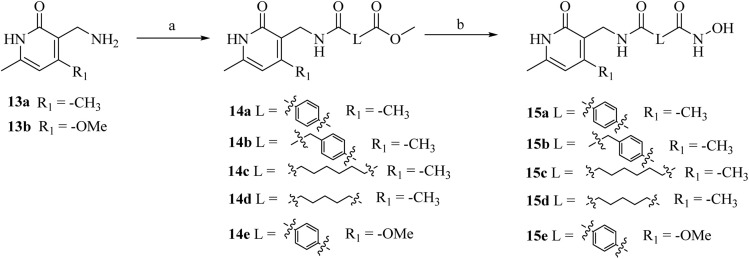
Reagents and conditions: (a) 2-(4-(methoxycarbonyl)phenyl)acetic acid; 4-(methoxycarbonyl)benzoic acid; 7-methoxy-7-oxoheptanoic acid or 8-methoxy-8-oxooctanoic acid; *N*,*N*-diisopropylethylamine (DIPEA); HATU; *N*,*N*-dimethylformamide (DMF), 0 °C, and 4 h and (b) NH_2_OH HCl, KOH, MeOH, 0 °C to r.t., and 6 h.

**Scheme 2:**
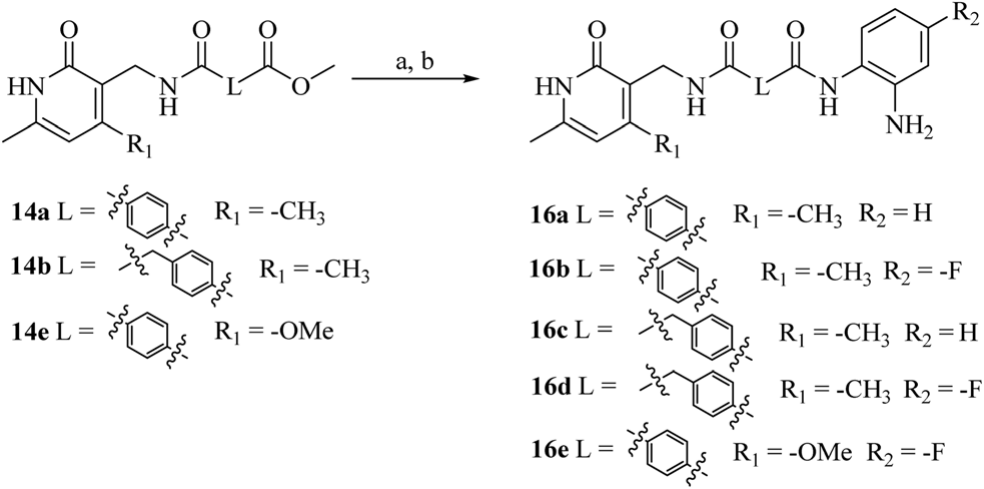
Reagents and conditions: (a) NaOH, MeOH/H_2_O, r.t., and 10 h and (b) HATU, DIPEA, DMF, *o*-phenylenediamine or 4-fluorobenzene-1, 2-diamine, r.t., and 6 h.

Hydroxamic acid analogs 15a–e were synthesized as shown in Scheme 1. The amidation of commercially available starting materials 13a–b with various linkers using 2-(7-aza-1*H*-benzotriazole-1-yl)-1,1,3,3-tetramethyluronium hexafluorophosphate (HATU) yielded intermediates 14a–e. Then, 14a–e were reacted with hydroxylamine to produce the corresponding target compounds 15a–e. *O*-phenylenediamine derivatives 16a–e were synthesized as depicted in Scheme 2. The hydrolysis of 14a, 14b and 14e yielded the corresponding carboxylic acids, and subsequent condensation with *o*-phenylenediamine or 4-fluorobenzene-1,2-diamine yielded 16a–e in high overall yields.

## 4 Results and discussion

### 4.1 HDAC1/6 and EZH2 inhibitory activities and SAR study of target compounds

All compounds were evaluated for HDAC1/6 inhibitory activities, with chidamide and nonselective SAHA taken as the positive controls (Table 1). Hydroxamic acid analogue 15c, bearing a six-carbon aliphatic chain linker, showed the highest potency against HDAC1 and HDAC6 (IC_50_ = 9.2 and 11.0 nM, respectively), whereas the five-carbon analogue 15d demonstrated reduced potency against both enzymes. Phenylhydroxamic acid-based 15a, with a 2-pyridone moiety linked to the phenyl linker *via* a carbonyl group, almost completely lost its HDAC1 activity and retained moderate HDAC6 inhibition. The insertion of an additional carbon atom between the carbonyl and phenyl linkers (15b) restored the inhibitory activity, with IC_50_ values of 1.36 µM against HDAC1 and 14.9 nM against HDAC6. These data indicated that an optimal linker length was essential for HDAC activity. Replacing the methyl substituent (15a) on 2-pyridone with a methoxy group (15e) did not improve the activity. *O*-phenylenediamine has not yet been used in reported dual EZH2/HDAC inhibitors. Compared to the corresponding hydroxamic acid analogues 15a and 15b, 16a and 16c exhibited significantly increased HDAC1 activity but not HDAC6 activity. 16c exhibited superior potency (IC_50_ of 138 nM), whereas the incorporation of a fluorine atom into the phenyl ring (16d) led to a slight decrease in HDAC1 activity. 16e, with a methoxy-substituted 2-pyridone as a capping group, also showed modest activity.

**Table 1:** Initial evaluation of the derivatives against HDAC1 and HDAC6 (IC_50_^a^, nM)

Compound	Structure	HDAC1	HDAC6
15a	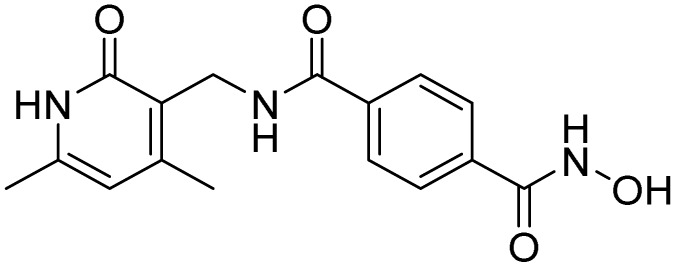	>10 000	68.0 ± 4.11
15b	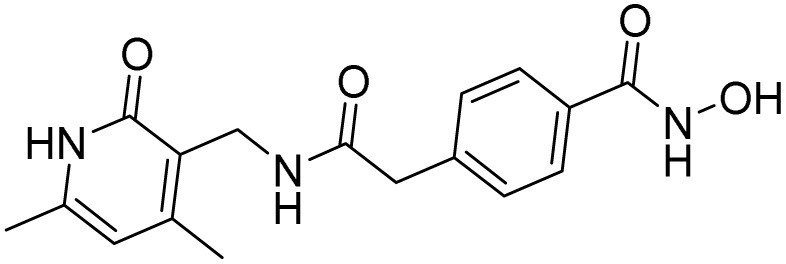	1360 ± 100	14.9 ± 1.20
15c	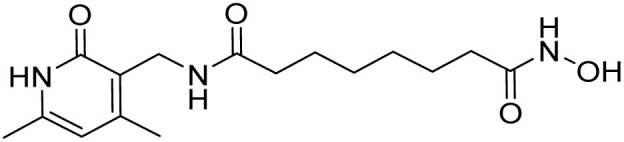	9.2 ± 0.40	11.0 ± 0.64
15d	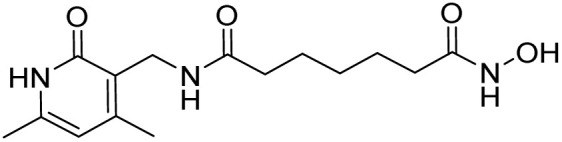	46.7 ± 2.30	23.5 ± 0.94
15e	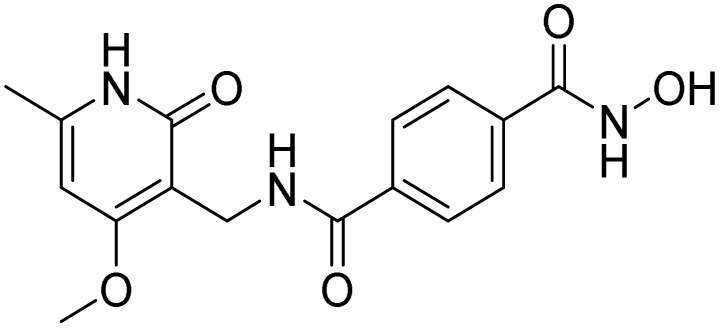	>10 000	96.0 ± 5.80
16a	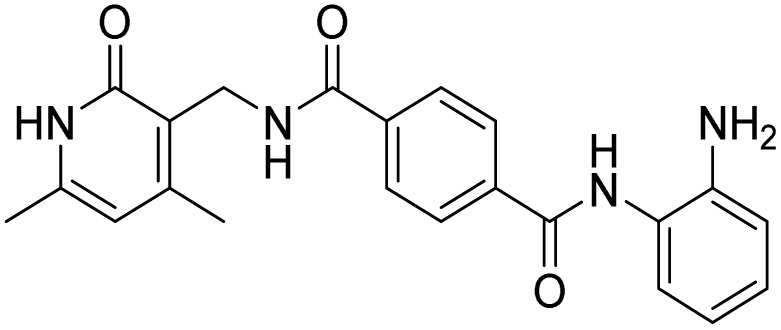	316 ± 18.6	>10 000
16b	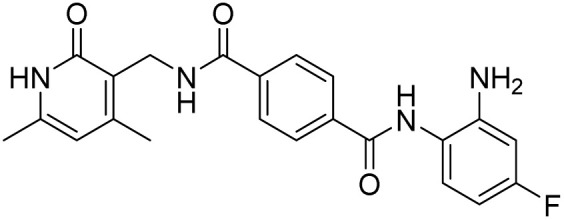	500 ± 39	>10 000
16c	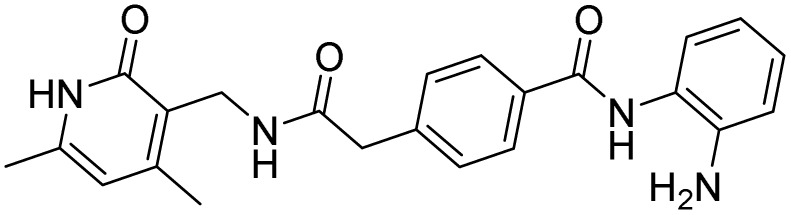	138 ± 8.20	>10 000
16d	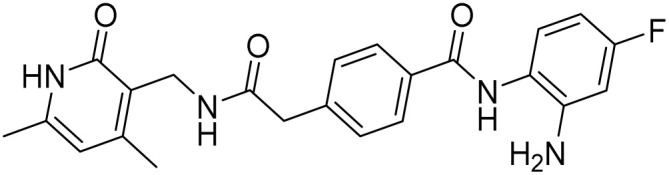	273 ± 15	>10 000
16e	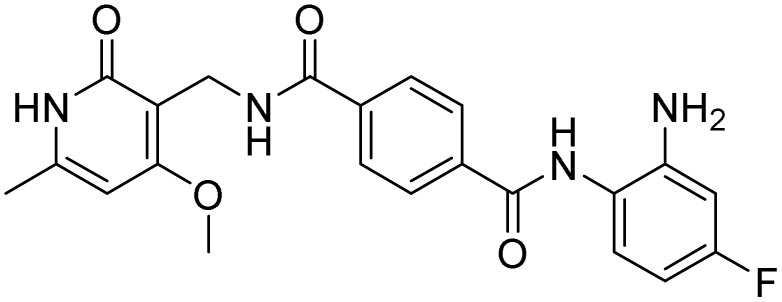	600 ± 33	>10 000
SAHA	—	4.30 ± 0.23	7.10 ± 0.15
Chidamide	—	95.4 ± 6.10	—

a IC_50_ values for the enzymatic inhibition of HDAC1 and HDAC6 enzymes. We performed experiments in duplicate, with SD < 15%. Assays were performed by the Reaction Biology Corporation (Malvern, PA, USA).

**Fig. 5:**
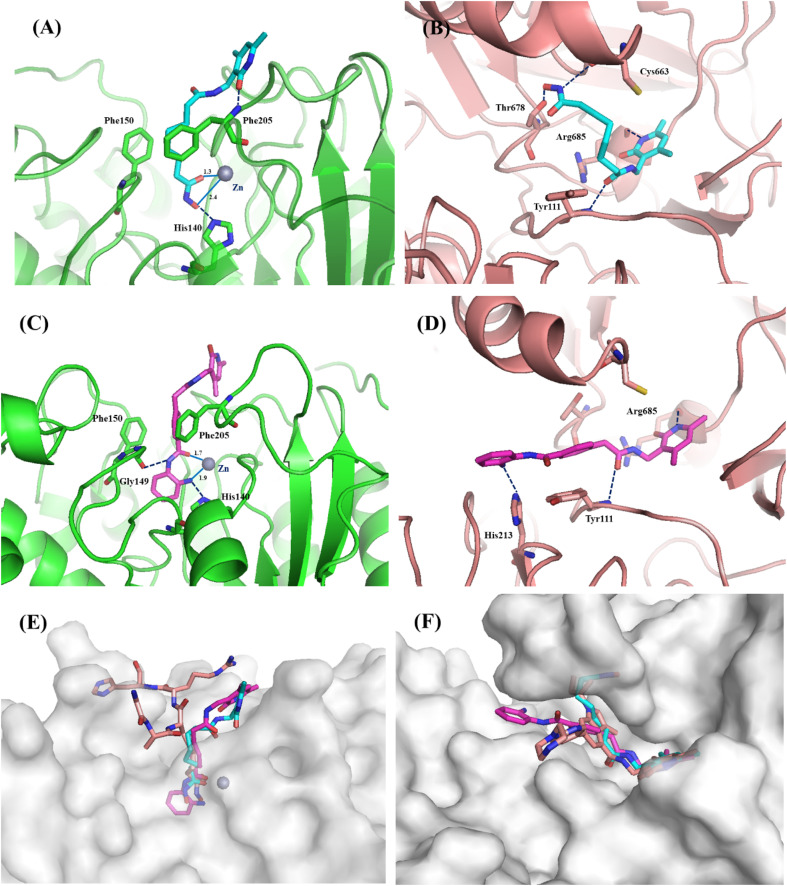
(A) Binding model of 15c (blue) in the catalytic pocket of human HDAC1 (PDB code: 5ICN). The Zn^2+^–O distances of 2.4 Å and 1.3 Å for the OH and C=O groups, respectively. (B) Binding model of 15c (blue) in the catalytic pocket of the human PRC2 crystal structure (PDB code: 5WG6). (C) Binding model of 16c (burgundy) in the catalytic pocket of human HDAC1. The Zn^2+^–O distances of 1.9 Å and 1.7 Å for the OH and C=O groups, respectively. (D) Binding model of 16c (burgundy) in the catalytic pocket of the human PRC2 crystal structure. (E) Overlay of 15c, 16c and the co-crystallized peptide inhibitor (pink) in HDAC1 (gray). (F) Overlay of 15c, 16c and the co-crystallized GSK126 inhibitor (pink) in the catalytic pocket of the human PRC2 crystal structure (gray). HDAC1 protein and PRC2 are labeled in green and light brown, respectively. The hydrogen bonds are labeled in dark blue (dashed line). Zinc ion is shown in brown. The distance between the functional group and zinc ion is represented by a blue solid line.

SAR analysis revealed several key determinants for HDAC inhibition. First, linker length proved to be critical: the six-carbon aliphatic chain was preferred, indicating that an optimal spacer length was essential for allowing the ZBG to coordinate well with the catalytic zinc ion at the bottom of the HDAC1 active site (as confirmed by the docking results in Fig. 5). Second, the ZBG played a pivotal role in determining isoform selectivity. *O*-phenylenediamine selectively inhibited HDAC1 over HDAC6. However, fluorination of the phenyl group of the ZBG slightly diminished HDAC1 activity, presumably due to the electron-withdrawing effect, which might impair the chelating ability of the ZBG.

15c and 16c were further screened for isoform selectivity against other HDACs. As depicted in Table 2, 15c inhibited HDAC2 and HDAC3 with IC_50_ values of 20.5 nM and 12.0 nM, respectively. 15c also showed weak inhibition against HDAC8 and 11 and exhibited no activity against HDAC4, 5 and 7. Its intracellular inhibitory activity was further confirmed by western blot analysis, which showed increased acetylation levels of histone H3 and tubulin (see Fig. S1 in SI). *O*-phenylenediamine derivative 16c selectively inhibited class I HDACs, with IC_50_ values of 138–235 nM against HDAC1-3. As shown in Table 3, 15c and 16c demonstrated submicromolar activity against wide and mutant EZH2 (Y641F). The superior activity of 16c suggested that the phenyl linker, a motif frequently employed in current EZH2 inhibitors, was more favorable. In summary, the SAR study revealed that the linker played a critical role in the enzyme inhibitory activity. An aliphatic chain enhanced HDAC1 inhibition, whereas a phenyl linker improved EZH2 binding.

**Table 2:** Complete characterization of 15c and 16c against other HDAC enzymes (IC_50_^a^, nM)

Compound	15c	16c	SAHA	Compound	15c	16c	SAHA
HDAC1	9.2 ± 0.40	138 ± 8.20	4.30 ± 0.23	HDAC6	11.0 ± 0.64	>10 000	7.10 ± 0.15
HDAC2	20.5 ± 1.40	210 ± 11	12.1 ± 1.28	HDAC7	>10 000	>10 000	>10 000
HDAC3	12.0 ± 0.86	235 ± 13	3.34 ± 0.27	HDAC8	1280 ± 77	1450 ± 94	1033 ± 63
HDAC4	>10 000	>10 000	>10 000	HDAC11	930 ± 80	>10 000	895 ± 72
HDAC5	>10 000	>10 000	>10 000				

a IC_50_ values for the enzymatic inhibition of HDAC enzymes. We performed experiments in duplicate, with SD < 15%. Assays were performed by the Reaction Biology Corporation (Malvern, PA, USA).

**Table 3:** EZH2 inhibitory activity of compounds 15c and 16c (IC_50_^a^, nM)

Compound	EZH2	EZH2^Y641F^
15c	311.1 ± 18	420.9 ± 21
16c	103.4 ± 9.4	124.1 ± 10
Tazemetostat	1.43 ± 0.36	7.03 ± 0.42

a IC_50_ values for EZH2 inhibition. We performed experiments in duplicate. Assays were performed by the Reaction Biology Corporation.

### 4.2 Molecular simulation

We docked 15c and 16c into the human HDAC1 crystal structure (PDB: 5ICN). As outlined in Fig. 5A, 15c could fit into the zinc-binding pocket, and the distance between the hydroxamic acid and the zinc ion suggested a possible bidentate chelation. Furthermore, the hydroxamic acid interacted with His140 at the bottom through a hydrogen bond. The 2-pyridone fragment was located at the cap region and formed a hydrogen bond with the Phe205 residue. 16c adopted a binding mode similar to that of 15c (Fig. 5C). The phenyl linker of 16c was inserted into the channel between Phe150 and Phe205 and formed π–π interactions. Besides, an additional hydrogen bond was observed between *o*-phenylenediamine and Gly149. As shown in Fig. 5E, 15c and 16c shared the same active pocket with the co-crystallized ligand (a peptide inhibitor) in HDAC1. Fig. 5B showed the docking pose of 15c with the human PRC2 crystal structure (PDB: 5WG6). The 2-pyridone core interacted with Arg685 through a hydrogen bond and formed H-bonds with Tyr111, Thr678 and Cys663. The aminomethylpyridone moiety of 16c also formed key hydrogen bonds with Arg685 and Tyr111 (Fig. 5D). Unlike the aliphatic chain, the phenyl linker of 16c formed π–π interactions with Tyr111. This extra interaction might enhance the binding affinity of 16c to the protein and resulted in a distinct spatial orientation of 16c. In addition, the amino group of *o*-phenylenediamine forms a hydrogen bond with His213. 15c and 16c also matched well with the co-crystallized ligand in EZH2 (Fig. 5F).

### 4.3 Antiproliferative activity evaluation

**Fig. 6:**
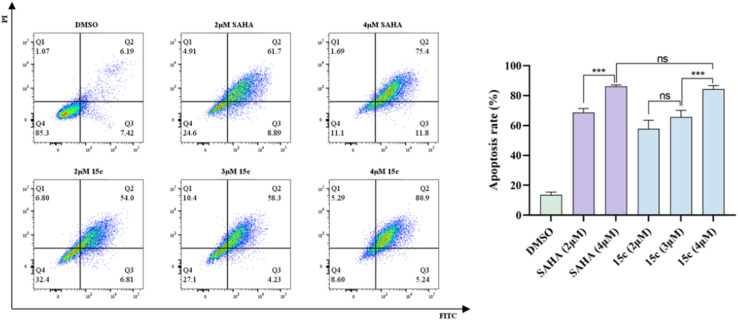
Induction of apoptosis at 48 h by compound 15c at 2, 3, and 4 µM concentrations in the MV4-11 cell line by flow cytometry analysis with SAHA (2, 4 µM) as a control. The percentage of cells in each part is indicated. Data are presented as the mean ± SD of three independent experiments (*n* = 3). Statistical significance was analyzed by one-way ANOVA; *p < 0.05, **p < 0.01, and ***p < 0.001 to indicate comparisons with the control group.

Clinically, most HDACis or EZH2 inhibitors are used for the treatment of hematologic malignancies such as leukemia and lymphoma. The synergistic effects of HDACis and EZH2 inhibitors at the cellular level have also been validated.^40–42^ Here, compounds with better enzyme activity were screened against the human AML cell line MV4-11 and DLBCL cell line SU-DHL-10. As shown in Table 4, 15c exhibited the best antiproliferative activity against both tumor cells (IC_50_ = 0.88 and 0.72 µM), comparable to that of SAHA, despite its weaker inhibitory activity against HDAC1-3. A similar result was observed for compound 16c and chidamide. 16c also exhibited potent antiproliferative activity against MV4-11 and SU-DHL-10 with IC_50_ values of 1.75 and 2.23 µM, respectively, which might be attributed to its dual inhibition. Besides, 15d and 16d exhibited single-digit micromolar antiproliferative activity, consistent with their enzymatic activities. For 15b, the lack of HDAC1 activity resulted in poor cellular activity. Notably, none of the compounds exhibited significant cytotoxicity against the normal bone marrow cell line, HS-5. The pro-apoptotic effects of these pyridones were further validated by flow cytometry. 15c induced MV4-11 cell apoptosis in a dose-dependent manner, achieving an apoptosis rate of 86.14% at 4 µM (Fig. 6). Moreover, the pharmacokinetic properties of 15c and 16c were predicted using the online platform SwissADME (https://swissadme.ch/). Both compounds complied with Lipinski's rule of five and were predicted to have good oral bioavailability (*F* = 0.55).

**Table 4:** Antiproliferative effects of 15b–d and 16c–d against MV4-11, SU-DHL-10 and HS-5 cell lines (IC_50_^a^, µM)

Compound	MV4-11	SU-DHL-10	HS-5
15b	18.2 ± 1.40	14.5 ± 1.22	>50
15c	0.88 ± 0.03	0.72 ± 0.04	>50
15d	7.60 ± 0.30	8.90 ± 0.66	>50
16c	1.75 ± 0.09	2.23 ± 0.10	>50
16d	3.90 ± 0.12	4.83 ± 0.16	>50
SAHA	0.93 ± 0.04	0.44 ± 0.02	—
Chidamide	2.80 ± 0.11	2.60 ± 0.20	—
Tazemetostat	23.0 ± 1.40	16.2 ± 0.36	—

a IC_50_ values are the averages of three independent experiments, with SD < 15%.

## 5 Conclusion

Combination therapy is a crucial treatment strategy for complex diseases such as cancer, but the associated drawbacks including unpredictable pharmacokinetics, drug–drug interactions, and poor patient compliance remain significant concerns. Therefore, the development of a single molecule inhibiting multiple targets offers a distinct advantage. By incorporating hydroxamic acid and *o*-phenylenediamine as ZBGs, we designed and synthesized ten novel pyridone derivatives as HDAC/EZH2 dual inhibitors. The best hydroxamic acid analog 15c exhibited excellent HDAC inhibition and antiproliferative activity at nanomolar concentrations, as well as moderate EZH2 activity. Apoptosis assay further validated its pro-apoptotic effect. Besides, as the first reported *o*-phenylenediamine dual inhibitor, 16c also exhibited single-digit micromolar antiproliferative activity against two cancer cell lines. More importantly, 16c could avoid the adverse effects associated with a hydroxamic acid moiety. In summary, the cellular activity profiles demonstrated that concurrent HDAC and EZH2 inhibition conferred a synergistic advantage. This combination may offer a superior therapeutic index, although additional in *vivo* assays are needed to support this conclusion. Currently, further structural modifications and biological investigations are under way in our laboratory.

## 6 Experimental section

### 6.1 Chemistry

All of the starting reagents were purchased and used with no additional purification. All of the mentioned yields were for isolated products. Melting points were determined in open capillaries on a WRS-1A digital melting point apparatus (Shenguang). ^1^H-NMR spectra were detected on a Bruker DRX-400 (400 MHz) using TMS as the internal standard. High-resolution mass spectra were obtained from Thermo Scientific Q Exactive. The chemical shifts are reported in ppm (*δ*), and the coupling constants (*J*) are given in Hertz (Hz). The purities of all target compounds were tested by HPLC and were found to be >95.0%. HPLC analysis was performed at room temperature using an Agilent Eclipse XDB-C18 (250 mm × 4.6 mm) column and plotted at 254 nm with 30% MeOH/H_2_O as the mobile phase.

#### 6.1.1 General procedure for the synthesis of intermediates 14a–e

Starting materials 13a or 13b (1 mmol) were added to a solution of DIPEA (0.66 mL, 4 mmol) and HATU (353.2 mg, 1.1 mmol) in DMF. After stirring for 30 min at 0 °C, 2-(4-(methoxycarbonyl)phenyl)acetic acid, 4-(methoxycarbonyl)benzoic acid, 7-methoxy-7-oxoheptanoic acid or 8-methoxy-8-oxooctanoic acid (1 mmol) was added. The mixture was stirred for an additional 4–6 h. Then, the mixture was poured into water and extracted with EtOAc. The combined organic extracts were washed with brine and concentrated *in vacuo*. The residues were purified by chromatography (petroleum ether : ethyl acetate = 4 : 1) on a silica gel column.

##### 6.1.1.1 Methyl 4-(((4,6-dimethyl-2-oxo-1,2-dihydropyridin-3-yl)methyl)carbamoyl)benzoate (14a)

White solid, yield 76.1%. ^1^H NMR (400 MHz, DMSO-*d*_6_) *δ* 11.50 (s, 1H), 8.57 (t, *J* = 4.9 Hz, 1H), 7.99 (d, *J* = 8.5 Hz, 2H), 7.94 (d, *J* = 8.5 Hz, 2H), 5.86 (s, 1H), 4.31 (d, *J* = 4.9 Hz, 2H), 3.86 (s, 3H), 2.17 (s, 3H), 2.11 (s, 3H).

##### 6.1.1.2 Methyl 4-(2-(((4,6-dimethyl-2-oxo-1,2-dihydropyridin-3-yl)methyl)amino)-2-oxoethyl)benzoate (14b)

White solid, yield 77.2%. ^1^H NMR (400 MHz, DMSO-*d*_6_) *δ* 10.67 (s, 1H), 7.27 (s, 1H), 7.04 (d, *J* = 8.3 Hz, 2H), 6.54 (d, *J* = 8.3 Hz, 2H), 5.00 (s, 1H), 3.23 (d, *J* = 5.1 Hz, 2H), 3.00 (s, 3H), 2.66 (s, 2H), 1.26 (s, 3H), 1.23 (s, 3H).

##### 6.1.1.3 Methyl 8-(((4,6-dimethyl-2-oxo-1,2-dihydropyridin-3-yl)methyl)amino)-8-oxooctanoate (14c)

White solid, yield 80.2%. ^1^H NMR (400 MHz, DMSO-*d*_6_) *δ* 11.51–11.42 (m, 1H), 7.67 (t, *J* = 5.1 Hz, 1H), 5.83 (s, 1H), 4.05 (d, *J* = 5.1 Hz, 2H), 3.57 (s, 3H), 2.26 (t, *J* = 7.4 Hz, 2H), 2.10 (d, *J* = 4.8 Hz, 6H), 2.02 (t, *J* = 7.4 Hz, 2H), 1.46–1.44 (m, 4H), 1.20–1.16 (m, 4H).

##### 6.1.1.4 *N*-hydroxy-4-(((5-(4-methoxyphenyl)-1,3, 4-oxadiazol-2-yl)thio)methyl)benzamide (14d)

White solid, yield 75.7%. ^1^H NMR (400 MHz, DMSO-*d*_6_) *δ* 11.52 (s, 1H), 10.40 (s, 1H), 8.70 (s, 1H), 7.68 (t, *J* = 5.1 Hz, 1H), 5.84 (s, 1H), 4.05 (d, *J* = 5.0 Hz, 2H), 2.10 (d, *J* = 2.0 Hz, 6H), 2.02 (t, *J* = 7.4 Hz, 2H), 1.91 (t, *J* = 7.4 Hz, 2H), 1.44–1.42 (m, 4H), 1.17–1.15 (m, 2H).

##### 6.1.1.5 Methyl 4-(((2-hydroxy-4-methoxy-6-methylpyridin-3-yl)methyl)carbamoyl)benzoate (14e)

White solid, yield 72.5%. ^1^H NMR (400 MHz, DMSO-*d*_6_) *δ* 11.46 (s, 1H), 8.39 (t, *J* = 4.5 Hz, 1H), 7.99 (d, *J* = 8.5 Hz, 2H), 7.92 (d, *J* = 8.5 Hz, 2H), 6.11 (s, 1H), 4.24 (d, *J* = 4.4 Hz, 2H), 3.87 (s, 3H), 3.80 (s, 3H), 2.19 (s, 3H).

#### 6.1.2 General procedure for the synthesis of target compounds 15a–e

To a solution of NH_2_OH HCl (1.70 g, 24.46 mmol) in MeOH (9 mL), KOH (1.70 g, 30.29 mmol) was added at 0 °C in an ice bath. Then, the mixture was stirred for 30 min and filtered. The esters from step 6.1.1 were added to the filtrate, and the reaction mixture was stirred for an additional 4 h at 0 °C. The resulting mixture was poured into water (30 mL), and the pH was adjusted to 7. The mixture was diluted with a saturated NaCl aqueous solution (40 mL) and extracted with EtOAc (50 mL × 3). After drying over Na_2_SO_4_, the organic phase was concentrated and purified by column chromatography (CH_2_Cl_2_ : MeOH = 10 : 1) to obtain the target compounds.

##### 6.1.2.1 *N* ^1^-((4, 6-dimethyl-2-oxo-1,2-dihydropyridin-3-yl)methyl)-*N*^4^-hydroxyterephthalamide (15a)

61.3% yield; white solid. Mp: 201.8–203.4 °C. ^1^H NMR (400 MHz, DMSO-*d*_6_) *δ* 11.50 (s, 1H), 8.58 (t, *J* = 4.9 Hz, 1H), 8.05–7.90 (m, 4H), 5.87 (s, 1H), 4.30 (d, *J* = 4.9 Hz, 2H), 3.87 (s, 2H), 2.17 (s, 3H), 2.11 (s, 3H). ^13^C NMR (400 MHz, DMSO-*d*_6_) *δ* 165.40, 163.42, 163.12, 149.79, 142.90, 136.69, 134.94, 127.41, 126.76, 121.48, 107.56, 35.48, 18.97, 18.24. HR-MS (ESI, *m*/*z*): calcd for 338.11113. C_16_H_17_N_3_NaO_4_^+^ [M + Na]^+^. Found 338.11136.

##### 6.1.2.2 4-(2-(((4,6-Dimethyl-2-oxo-1,2-dihydropyridin-3-yl)methyl)amino)-2-oxoethyl)-*N*-hydroxybenzamide (15b)

63.7% yield; white solid. Mp: 164.3–166.1 °C. ^1^H NMR (400 MHz, DMSO-*d*_6_) *δ* 11.52 (s, 1H), 11.22 (s, 1H), 9.19 (s, 1H), 8.07 (t, *J* = 5.2 Hz, 1H), 7.65 (d, *J* = 8.3 Hz, 2H), 7.29 (d, *J* = 8.1 Hz, 2H), 5.83 (s, 1H), 4.07 (d, *J* = 5.0 Hz, 2H), 3.45 (s, 2H), 2.10 (s, 3H), 2.08 (s, 3H). ^13^C NMR (101 MHz, DMSO-*d*_6_) *δ* 169.35, 164.09, 163.07, 149.48, 142.91, 139.98, 130.87, 128.99, 126.75, 121.86, 107.42, 41.85, 34.50, 18.83, 18.22. HR-MS (ESI, *m*/*z*): calcd for 352.12678. C_17_H_19_N_3_NaO_4_^+^ [M + Na]^+^. Found 352.12653.

##### 6.1.2.3 *N* ^1^-((4,6-dimethyl-2-oxo-1,2-dihydropyridin-3-yl)methyl)-*N*^8^-hydroxyoctanediamide (15c)

58.6% yield; white solid. Mp: 194.5–206.2 °C. ^1^H NMR (400 MHz, DMSO-*d*_6_) *δ* 11.43 (s, 1H), 10.30 (s, 1H), 8.60 (s, 1H), 7.69 (t, *J* = 5.1 Hz, 1H), 5.83 (s, 1H), 4.05 (d, *J* = 5.1 Hz, 2H), 2.10 (d, *J* = 2.4 Hz, 6H), 2.02 (t, *J* = 7.4 Hz, 2H), 1.91 (t, *J* = 7.4 Hz, 2H), 1.51–1.37 (m, 4H), 1.18 (dd, *J* = 7.2, 3.6 Hz, 4H). ^13^C NMR (101 MHz, DMSO-*d*_6_) *δ* 171.87, 169.17, 163.05, 149.40, 142.76, 122.05, 107.44, 35.06, 34.15, 32.24, 28.42, 28.39, 25.30, 25.08, 18.85, 18.21. HR-MS (ESI, *m*/*z*): calcd for 346.17373. C_16_H_25_N_3_NaO_4_^+^ [M + Na]^+^. Found 346.17368.

##### 6.1.2.4 *N* ^1^-((4,6-dimethyl-2-oxo-1,2-dihydropyridin-3-yl)methyl)-*N*^7^-hydroxyheptanediamide (15d)

67.2% yield; white solid. Mp: 190.2–192.2 °C. ^1^H NMR (400 MHz, DMSO-*d*_6_) *δ* 11.52 (s, 1H), 10.40 (s, 1H), 8.70 (s, 1H), 7.68 (t, *J* = 5.1 Hz, 1H), 5.84 (s, 1H), 4.05 (d, *J* = 5.0 Hz, 2H), 2.10 (d, *J* = 2.0 Hz, 6H), 2.02 (t, *J* = 7.4 Hz, 2H), 1.91 (t, *J* = 7.4 Hz, 2H), 1.43 (pd, *J* = 7.4, 4.2 Hz, 4H), 1.16 (td, *J* = 8.4, 4.2 Hz, 2H). ^13^C NMR (101 MHz, DMSO-*d*_6_) *δ* 171.94, 169.16, 163.13, 149.59, 142.85, 122.05, 107.59, 35.02, 34.23, 32.20, 28.30, 25.18, 25.00, 18.90, 18.26. HR-MS (ESI, *m*/*z*): calcd for 332.15808. C_15_H_23_N_3_NaO_4_^+^ [M + Na]^+^. Found 332.15793.

##### 6.1.2.5 *N* ^1^-hydroxy-*N*^4^-((4-methoxy-6-methyl-2-oxo-1,2-dihydropyridin-3-yl)methyl)terephthalamide (15e)

45.8% yield; white solid. Mp: 174.3–175.8 °C. ^1^H NMR (400 MHz, DMSO-*d*_6_) *δ* 11.47 (s, 1H), 11.33 (s, 1H), 9.16 (s, 1H), 8.31 (s, 1H), 7.86 (d, *J* = 8.0 Hz, 2H), 7.78 (d, *J* = 8.1 Hz, 2H), 6.11 (s, 1H), 4.24 (d, *J* = 4.4 Hz, 2H), 3.80 (s, 3H), 2.19 (s, 3H). ^13^C NMR (101 MHz, DMSO-*d*_6_) *δ* 166.08, 165.25, 163.91, 145.88, 136.90, 134.84, 129.08, 127.30, 126.78, 105.08, 93.56, 56.14, 33.32, 18.84. HR-MS (ESI, *m*/*z*): calcd for 354.10604. C_16_H_17_N_3_NaO_5_^+^ [M + Na]^+^. Found 354.10587.

#### 6.1.3 General procedure for the synthesis of target compounds 16a–e

Compounds were synthesized *via* a two-step, one-pot process: (i) to a solution of various esters (1 mmol) in MeOH and H_2_O (10 mL, v/v = 1 : 1), NaOH was added (160 mg, 4 mmol) at 0 °C. Then, the mixture was allowed to stir at room temperature for 10 h. The reaction was monitored by TLC until the starting material was consumed. After neutralization with acetic acid, the precipitate was collected by filtration, washed with water, and dried *in vacuo*. (ii) To a solution of acid (1 mmol) from step i, DIPEA was added (0.66 mL, 4 mmol), HATU (353.2 mg, 1.1 mmol) and DMF (10 mL) at 0 °C. After 30 min, various amides (1 mmol) were added. The mixture was stirred for 6 h. After the reaction was complete, the mixture was poured into water (30 mL) and extracted with EtOAc (3 × 30 mL). The combined organic extracts were washed with brine (2 × 30 mL), dried over anhydrous Na_2_SO_4_ and concentrated *in vacuo*. The product was obtained by chromatography (CH_2_Cl_2_ : MeOH = 20 : 1) on a silica gel column.

##### 6.1.3.1 *N* ^1^-(2-aminophenyl)-*N*^4^-((4, 6-dimethyl-2-oxo-1,2-dihydropyridin-3-yl)methyl)terephthalamide (16a)

79.2% yield; white solid. Mp: 156.2–158.0 °C. ^1^H NMR (400 MHz, DMSO-*d*_6_) *δ* 11.50 (s, 1H), 9.76 (s, 1H), 8.54 (d, *J* = 5.1 Hz, 1H), 8.02 (d, *J* = 8.3 Hz, 2H), 7.95 (d, *J* = 8.3 Hz, 2H), 7.16 (d, *J* = 7.7 Hz, 1H), 6.97 (t, *J* = 7.6 Hz, 1H), 6.78 (d, *J* = 7.6 Hz, 1H), 6.59 (t, *J* = 7.4 Hz, 1H), 5.88 (s, 1H), 4.92 (s, 2H), 4.32 (d, *J* = 4.9 Hz, 2H), 2.19 (s, 3H), 2.12 (s, 3H). ^13^C NMR (101 MHz, DMSO-*d*_6_) *δ* 165.36, 164.77, 163.10, 149.73, 143.29, 142.87, 136.79, 136.72, 127.67, 127.25, 126.83, 126.66, 123.04, 121.48, 116.20, 116.09, 107.51, 35.51, 18.96, 18.21. HR-MS (ESI, *m*/*z*): calcd for 413.15841. C_22_H_22_N_4_NaO_3_^+^ [M + Na]^+^. Found 413.15842.

##### 6.1.3.2 *N* ^1^-(2-amino-4-fluorophenyl)-*N*^4^-((4,6-dimethyl-2-oxo-1,2-dihydropyridin-3-yl)methyl)terephthalamide (16b)

57.8% yield; white solid. Mp: 166.6–168.3 °C. ^1^H NMR (400 MHz, DMSO-*d*_6_) *δ* 11.50 (s, 1H), 9.68 (s, 1H), 8.53 (t, *J* = 5.0 Hz, 1H), 8.01 (d, *J* = 8.3 Hz, 2H), 7.94 (d, *J* = 8.2 Hz, 2H), 7.10 (dd, *J* = 8.7, 6.3 Hz, 1H), 6.53 (dd, *J* = 11.3, 2.9 Hz, 1H), 6.35 (td, *J* = 8.5, 2.9 Hz, 1H), 5.88 (s, 1H), 5.26 (s, 2H), 4.32 (d, *J* = 4.9 Hz, 2H), 2.19 (s, 3H), 2.12 (s, 3H). ^13^C NMR (101 MHz, DMSO-*d*_6_) *δ* 165.36, 165.06, 163.10, 159.95, 149.74, 145.62, 142.88, 136.80, 136.60, 128.64, 127.70, 127.22, 121.47, 119.04, 107.52, 35.52, 18.96, 18.21. HR-MS (ESI, *m*/*z*): calcd for 431.14899. C_22_H_21_FN_4_NaO_3_^+^ [M + Na]^+^. Found 431.14896.

##### 6.1.3.3 *N*-(2-aminophenyl)-4-(2-(((4,6-dimethyl-2-oxo-1,2-dihydropyridin-3-yl)methyl)amino)-2-oxoethyl)benzamide (16c)

61.7% yield; white solid. Mp: 181.0–183.2 °C. ^1^H NMR (400 MHz, DMSO-*d*_6_) *δ* 11.52 (s, 1H), 9.61 (s, 1H), 8.08 (t, *J* = 5.2 Hz, 1H), 7.88 (d, *J* = 7.9 Hz, 2H), 7.36 (d, *J* = 8.0 Hz, 2H), 7.16 (dd, *J* = 7.9, 1.5 Hz, 1H), 6.96 (td, *J* = 7.5, 1.6 Hz, 1H), 6.77 (dd, *J* = 8.0, 1.5 Hz, 1H), 6.59 (td, *J* = 7.5, 1.5 Hz, 1H), 5.85 (s, 1H), 4.90 (s, 2H), 4.08 (d, *J* = 5.1 Hz, 2H), 3.49 (s, 2H), 2.10 (d, *J* = 4.9 Hz, 6H). ^13^C NMR (101 MHz, DMSO-*d*_6_) *δ* 169.41, 165.27, 163.08, 149.54, 143.13, 142.95, 140.31, 132.72, 128.91, 127.69, 126.70, 126.48, 123.42, 121.86, 116.30, 116.17, 107.47, 41.94, 34.54, 18.85, 18.23. HR-MS (ESI, *m*/*z*): calcd for 427.17406. C_23_H_24_N_4_NaO_3_^+^ [M + Na]^+^. Found 427.17404.

##### 6.1.3.4 *N*-(2-amino-4-fluorophenyl)-4-(2-(((4,6-dimethyl-2-oxo-1,2-dihydropyridin-3-yl)methyl)amino)-2-oxoethyl)benzamide (16d)

63.1% yield; white solid. Mp: 163.0–164.5 °C. ^1^H NMR (400 MHz, DMSO-*d*_6_) *δ* 11.52 (s, 1H), 9.54 (s, 1H), 8.07 (t, *J* = 5.1 Hz, 1H), 7.88 (d, *J* = 7.9 Hz, 2H), 7.35 (d, *J* = 7.9 Hz, 2H), 7.11 (dd, *J* = 8.7, 6.3 Hz, 1H), 6.53 (dd, *J* = 11.2, 2.9 Hz, 1H), 6.35 (td, *J* = 8.5, 2.9 Hz, 1H), 5.84 (s, 1H), 5.23 (s, 2H), 4.08 (d, *J* = 5.1 Hz, 2H), 3.48 (s, 2H), 2.10 (d, *J* = 5.0 Hz, 6H). ^13^C NMR (101 MHz, DMSO-*d*_6_) *δ* 169.34, 163.04, 149.44, 145.39, 142.90, 140.31, 132.57, 128.84, 127.68, 121.86, 107.38, 53.59, 41.91, 34.50, 18.82, 18.09, 16.74. HR-MS (ESI, *m*/*z*): calcd for 445.16464. C_23_H_23_FN_4_NaO_3_^+^ [M + Na]^+^. Found 445.16443.

##### 6.1.3.5 *N* ^1^-(2-amino-4-fluorophenyl)-*N*^4^-((4-methoxy-6-methyl-2-oxo-1,2-dihydropyridin-3-yl)methyl)terephthalamide (16e)

59.3% yield; white solid. Mp: 157.0–158.5 °C. ^1^H NMR (400 MHz, DMSO-*d*_6_) *δ* 11.49 (s, 1H), 9.70 (s, 1H), 8.37 (t, *J* = 4.5 Hz, 1H), 8.02 (d, *J* = 8.4 Hz, 2H), 7.92 (d, *J* = 8.3 Hz, 2H), 7.11 (dd, *J* = 8.7, 6.3 Hz, 1H), 6.54 (dd, *J* = 11.2, 2.9 Hz, 1H), 6.35 (td, *J* = 8.5, 2.9 Hz, 1H), 6.13 (s, 1H), 5.27 (s, 2H), 4.26 (d, *J* = 4.4 Hz, 2H), 3.81 (s, 3H), 2.20 (s, 3H). ^13^C NMR (101 MHz, DMSO-*d*_6_) *δ* 166.04, 165.19, 165.06, 163.86, 145.86, 145.66, 145.54, 136.97, 128.62, 127.70, 127.10, 119.02, 105.06, 102.09, 101.53, 93.53, 62.80, 56.13, 33.34, 18.82. HR-MS (ESI, *m*/*z*): calcd for 447.14390. C_22_H_21_FN_4_NaO_4_^+^ [M + Na]^+^. Found 447.14386.

### 6.2 *In vitro* HDAC enzyme assay

The IC_50_ testing of the compounds was performed by the Reaction Biology Corporation. Detailed methods can be found in our previously published literature.^13,53^

### 6.3 EZH2 inhibition assay

The IC_50_ testing of the compounds was performed by the Reaction Biology Corporation. Detailed methods can be found at https://www.reactionbiology.com/datasheet/ezh2_complex_methyl_malvern/.

### 6.4 Cell culture and antiproliferative assay

All cells (obtained from Procell Life Science & Technology Co., Ltd) were maintained at 37 °C in a humidified atmosphere of 5% CO_2_ in air. The cells were cultured in IMDM medium with 20% FBS, 100 U mL^−1^ penicillin and 100 µg mL^−1^ streptomycin. Briefly, a 100 µL volume of cell suspension or complete medium was plated into a 96-well plate (5 × 10^4^ cells per well) for 24 h. The compounds were serially diluted to concentrations of 10, 5, 2.5, 1.25, 0.625, and 0.375 µM and incubated for 48 h. Then, an Alamar blue solution (10%v/v) was pipetted into each well of the 96-well plate; and the plate was incubated for an additional 5–6 h. The absorbance (OD) was measured at 530/590 nm. Data were normalized to the vehicle groups (DMSO) and are represented as the means of three independent measurements with standard errors of <20%. The IC_50_ values were calculated using Prism 5.0.

### 6.5 Apoptosis assay

For the flow cytometry assay, detailed methods can be found in our previously published literature.^54^

### 6.6 Computational methods

Molecular docking was performed using the SYBYL-X 2.0 software (222 S Central Ave Ste 1008, Saint Louis, MO 63105, USA). This software could reproduce the binding mode of the co-crystallized ligands in HDAC1 or EZH2, with RMSD values of <2 Å (see Fig. S2 in the SI). For HDAC1, the cocrystal of PDB: 5ICN was used for docking. The peptide inhibitor GLY-ALA-6A0-ARG-HIS was selected as the ligand binding site. For human PRC2, the cocrystal of PDB: 5WG6 was used for docking. 1-[(2*S*)-butan-2-yl]-*N*-[(4,6-dimethyl-2-oxo-1,2-dihydropyridin-3-yl)methyl]-3-methyl-6-[6-(piperazin-1-yl)pyridin-3-yl]-1*H*-indole-4-carboxamide was selected as the ligand binding site. The water molecules outside the binding pocket were excluded. The other docking parameters were set to default.

## Author contributions

Conceptualisation: Xin Chen and Qiang Yu; methodology: Zhaobang Tan and Jiajun Ding; validation: Lei Qian; formal analysis: Zhaobang Tan and Lei Qian; writing – original draft preparation: Qiang Yu and Lei Qian; and writing – review and editing: Xin Chen. All authors have read and agreed to the published version of the manuscript.

## Conflicts of interest

The authors declare that they have no known competing financial interests or personal relationships that could have appeared to influence the work reported in this paper.

## Supplementary Material

RA-OLF-D6RA04928A-s001

## Data Availability

Data can be made available on reasonable request to the corresponding author. All data pertaining to this article are included within the manuscript and its supplementary information (SI). Supplementary information is available. See DOI: https://doi.org/10.1039/d6ra04928a.
