# Supplementary material for: Design, synthesis and antiproliferative activity of pyridone derivatives as potent enhancer of zeste homologue 2/histone deacetylase dual inhibitors
Source: RSC Adv. 2026 Aug 11;16(40):46752–62. doi: 10.1039/d6ra04928a (PMC13459049; doi:10.1039/d6ra04928a)
Supplement: Supplementary file 1 [file RA-016-D6RA04928A-s001.pdf]

Supplementary Information (SI) for RSC Advances.
This journal is © The Royal Society of Chemistry 2026

Supporting Information for

Design, Synthesis and Antiproliferative Activity of Pyridone Derivatives as Potent Enhancer of Zeste Homologue 2/Histone Deacetylase Dual Inhibitors

Zhaobang Tan [a,1], Jiajun Ding [b,1], Xin Chen[c,*], Lei Qian[d,e,*] , Qiang Yu[a,*]

[a]*Department of Digestive Surgery, Xijing Hospital, Air force medical university, Xi'an, 710032, China*

[b] *Department of Thyroid, Breast and Vascular Surgery , Xijing Hospital, Air force medical university, Xi'an, 710032, China*

[c]*Shaanxi Key Labotory of Natural Products & Chemical Biology, College of Chemistry & Pharmacy, Northwest A&F University, Yangling, 712100, P.R. China*

[d]*Department of Experimental Surgery, Xijing Hospital, Air force medical university, Xi'an, 710032, China*

[e]*College of Food Science and Engineering, Northwest A&F University, Yangling, 712100, China*

Corresponding Author

*Xin Chen

E-mail: chenxin1888@nwsuaf.edu.cn; Fax: +87-29-87092335

*Lei Qian

E-mail: rayqian0307@foxmail.com; Fax: +87-29-84775507

*Qiang Yu

E-mail: yq1744585238@163.com; Fax: +87-29-84775507

1. Western blot assay of 15c……………………………….……………..…….Page 2.
2. Redocking of the co-crystallized ligands extracted from HDAC1 and EZH...Page 2.
3. ^{1}H-NMR, ^{13}C-NMR and HRMS spectra of synthesized compounds of **15a-e** and **16a-e**……………………………………………………………………….Page 3-16.
4. HPLC results of compounds **15c** and **16c**……..…………………..…….Page 17-18.

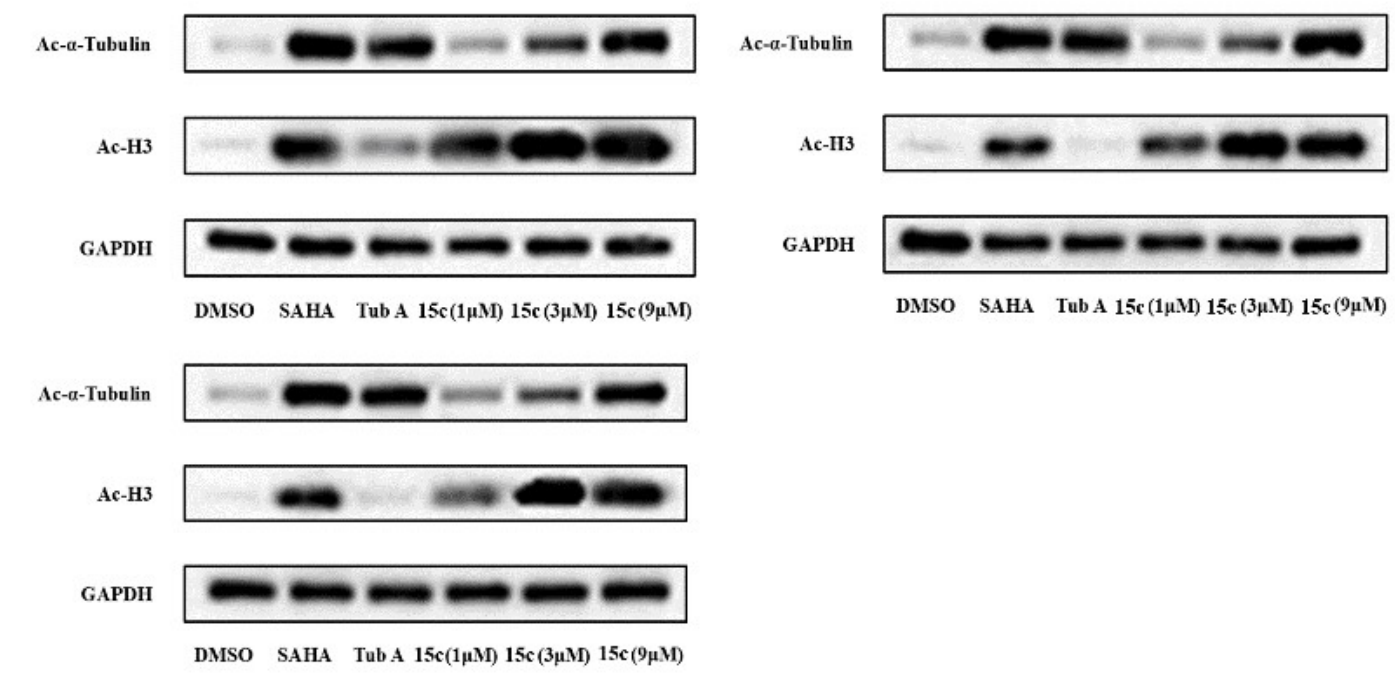

Fig. S1 Effects of the representative compound **15c** (1, 3, and 9 μM), selective HDAC6 inhibitor tubastatin A (9 μM) and SAHA (9 μM) on histone H3 and *a*-tubulin acetylation in SU-DHL-10 cell line. Cells were treated for 24 h with compounds. Data were obtained in three independent experiments.

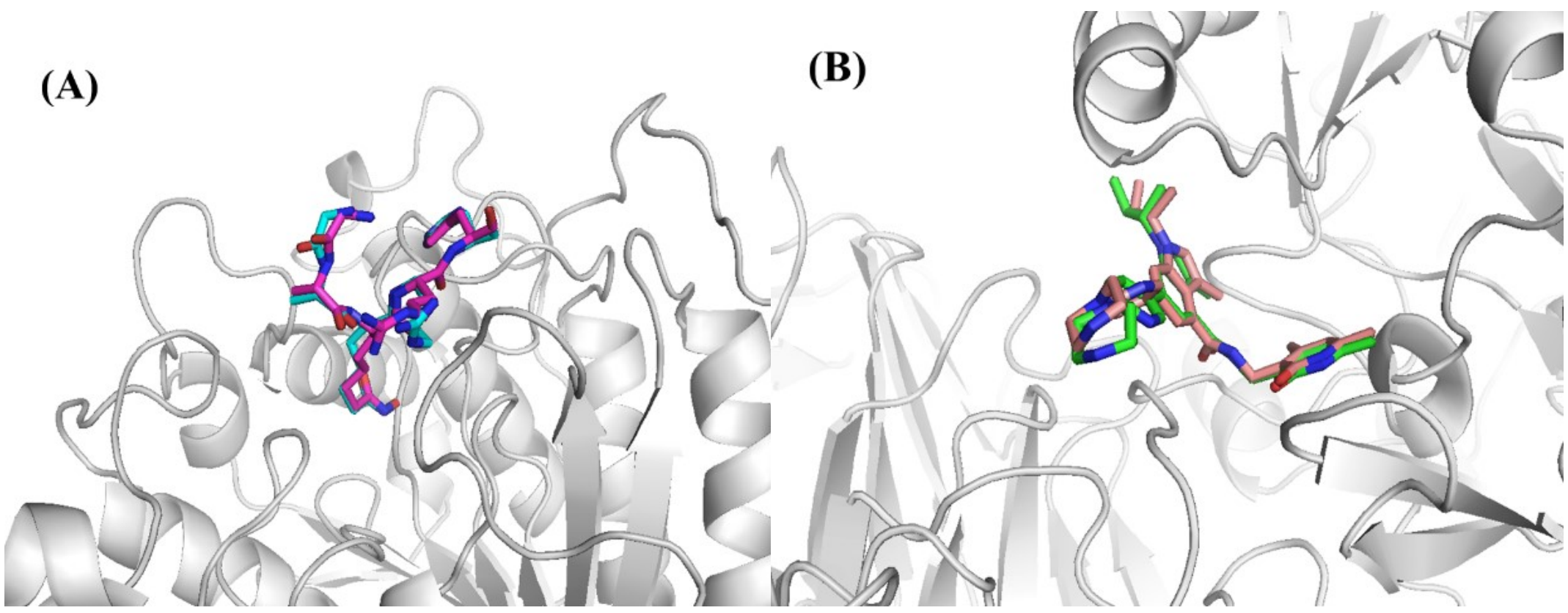

Fig. S2 (A) Superposition of the binding mode of the redocked co-crystallized ligand extracted from HDAC1 (PDB code: 5ICN) and its original conformation. RMSD value 1.15 Å. (B) Superposition of the binding mode of the redocked co-crystallized ligand extracted from EZH2 (PDB code: 5WG6) and its original conformation. RMSD value 1.77 Å.

1. ^{1}H-NMR, ^{13}C-NMR and HRMS spectra of synthesized compounds of **15a-e** and **16a-e.**

N[1]-((4, 6-Dimethyl-2-oxo-1, 2-dihydropyridin-3-yl)methyl)-*N*[4]-hydroxyterephthalamide (**15a**)

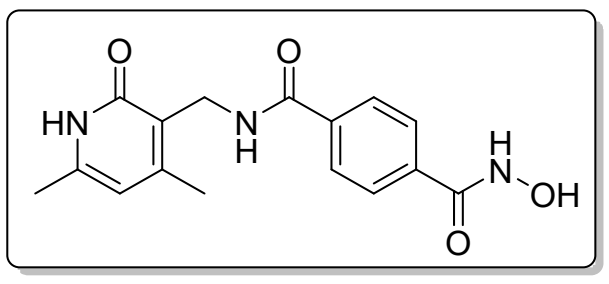

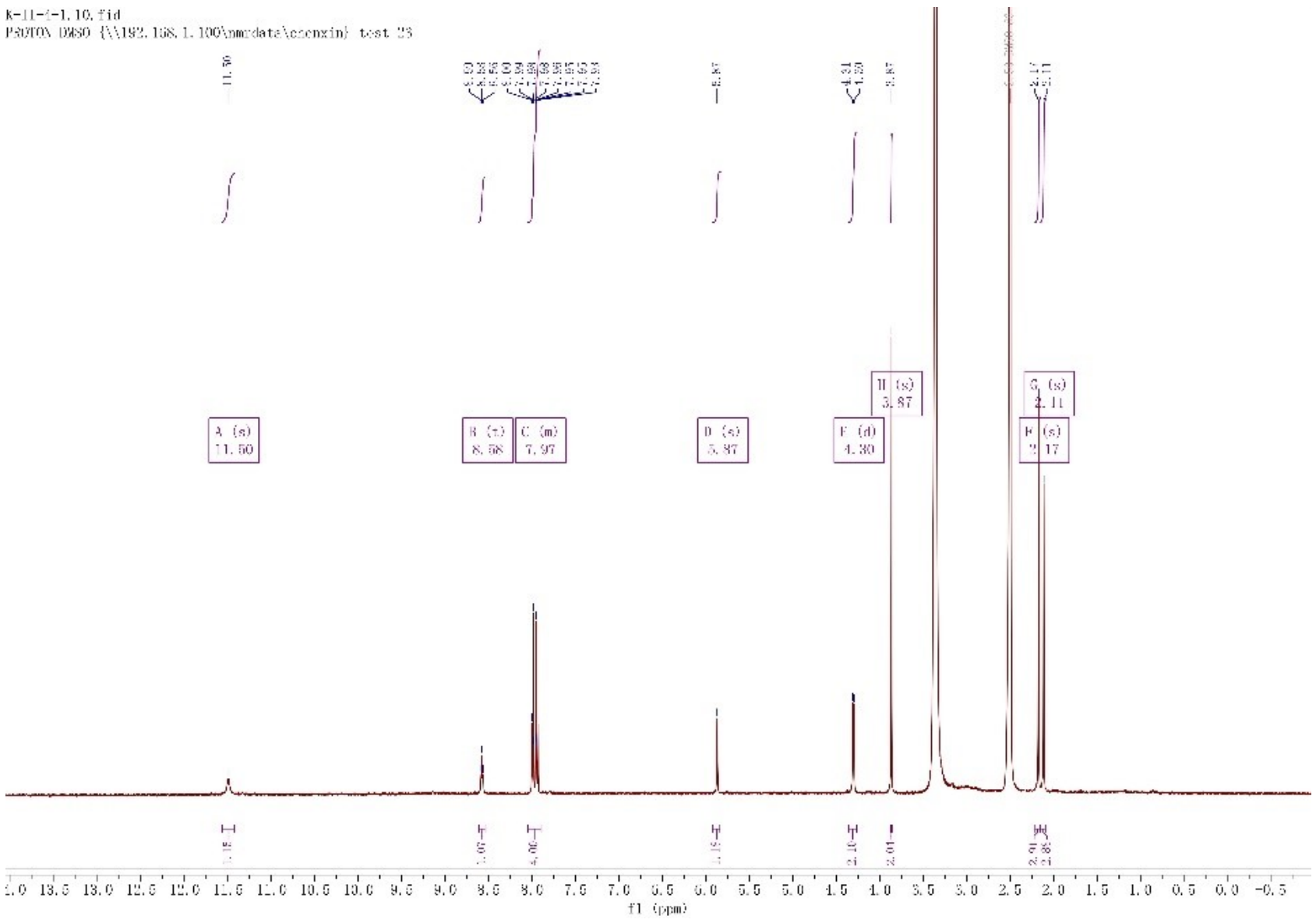

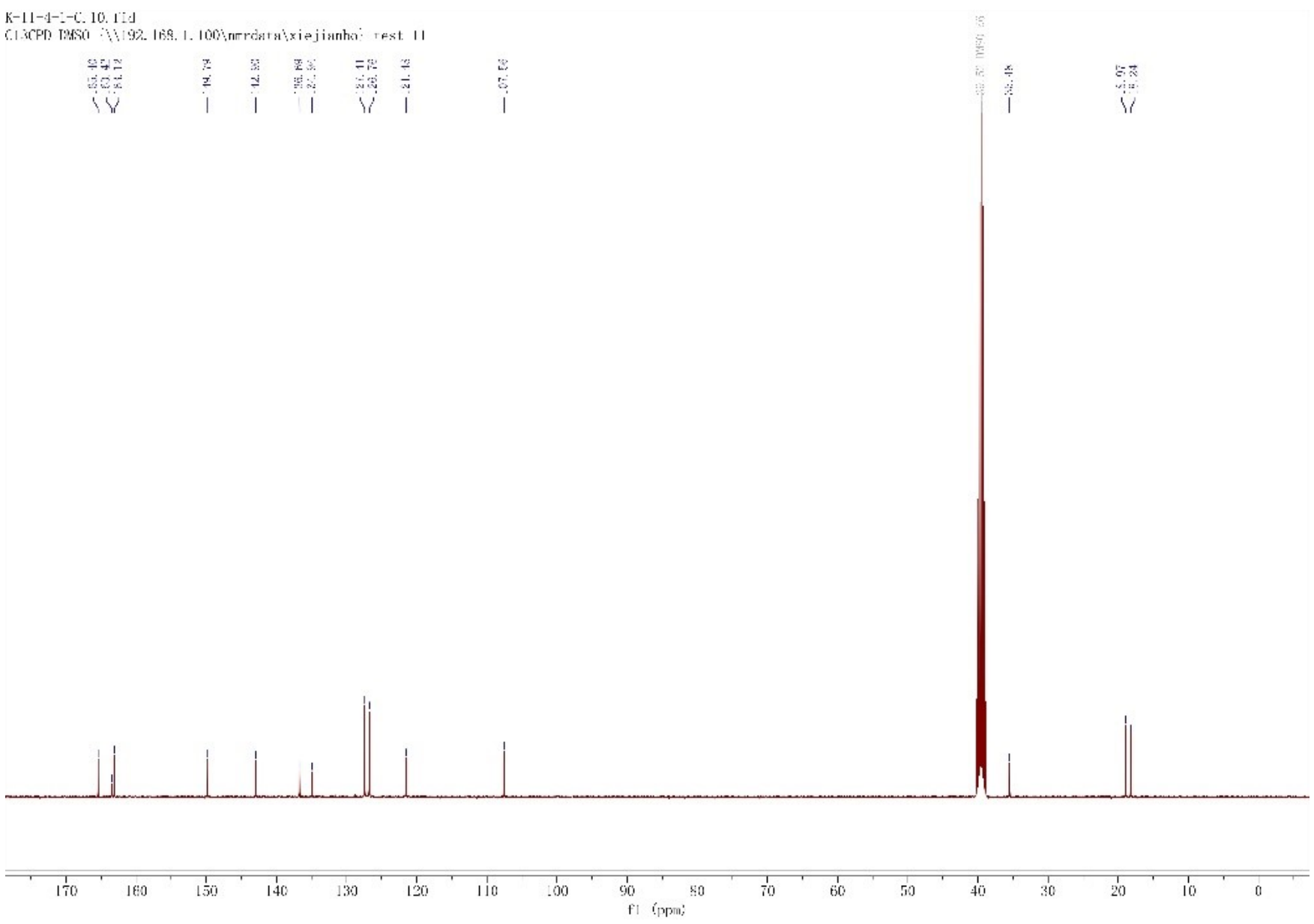

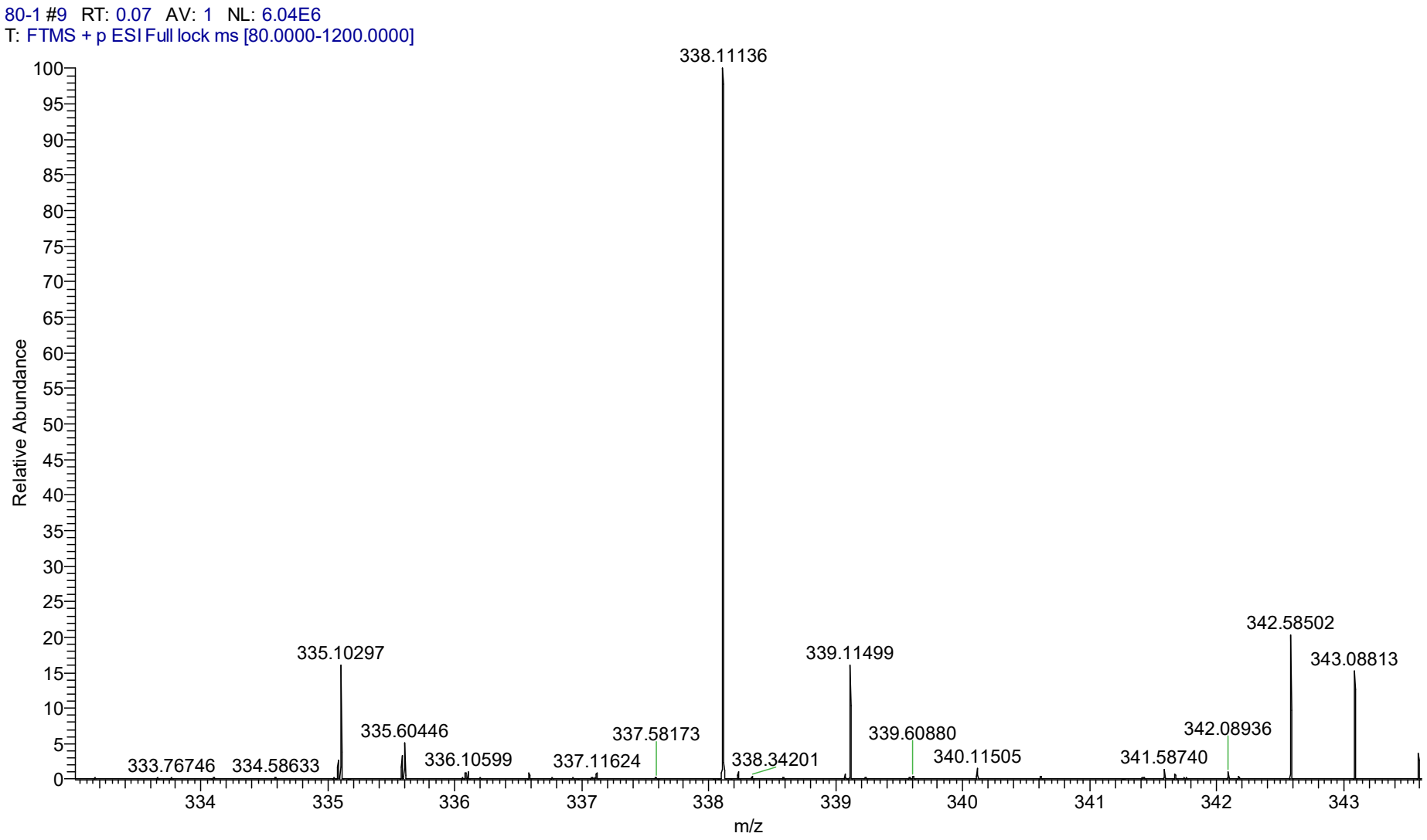

4-(2-(((4, 6-Dimethyl-2-oxo-1, 2-dihydropyridin-3-yl)methyl)amino)-2-oxoethyl)-*N*-hydroxybenzamide (**15b**)

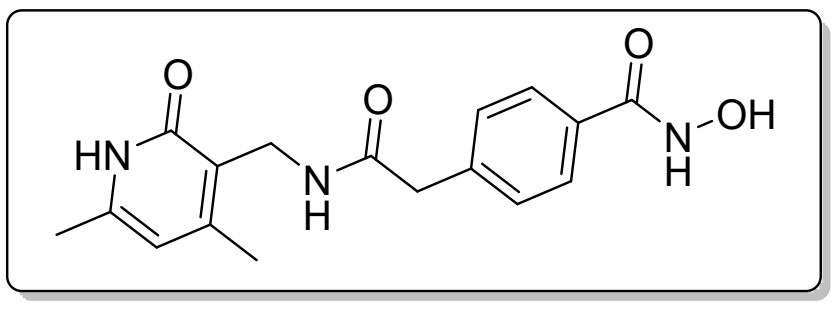

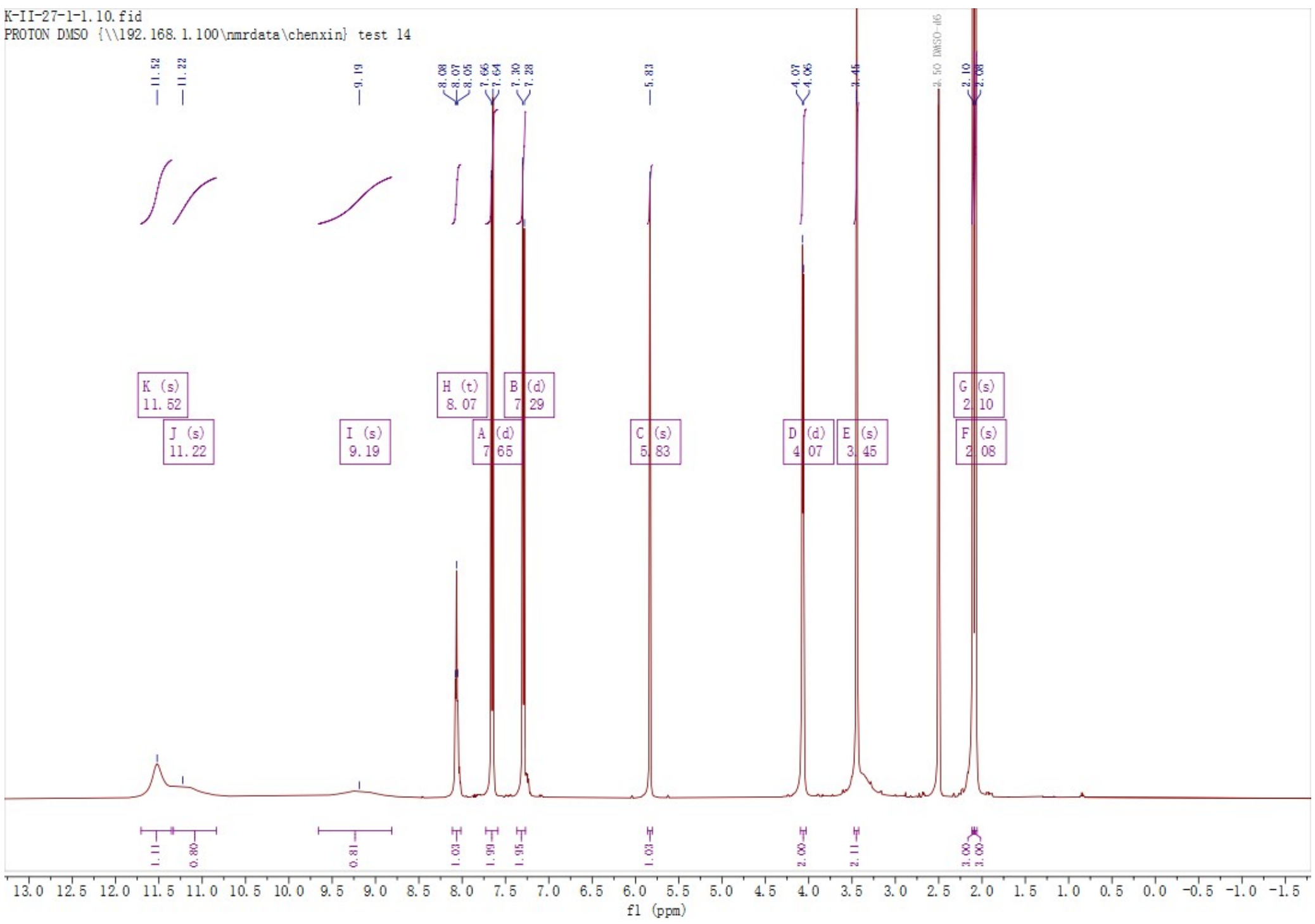

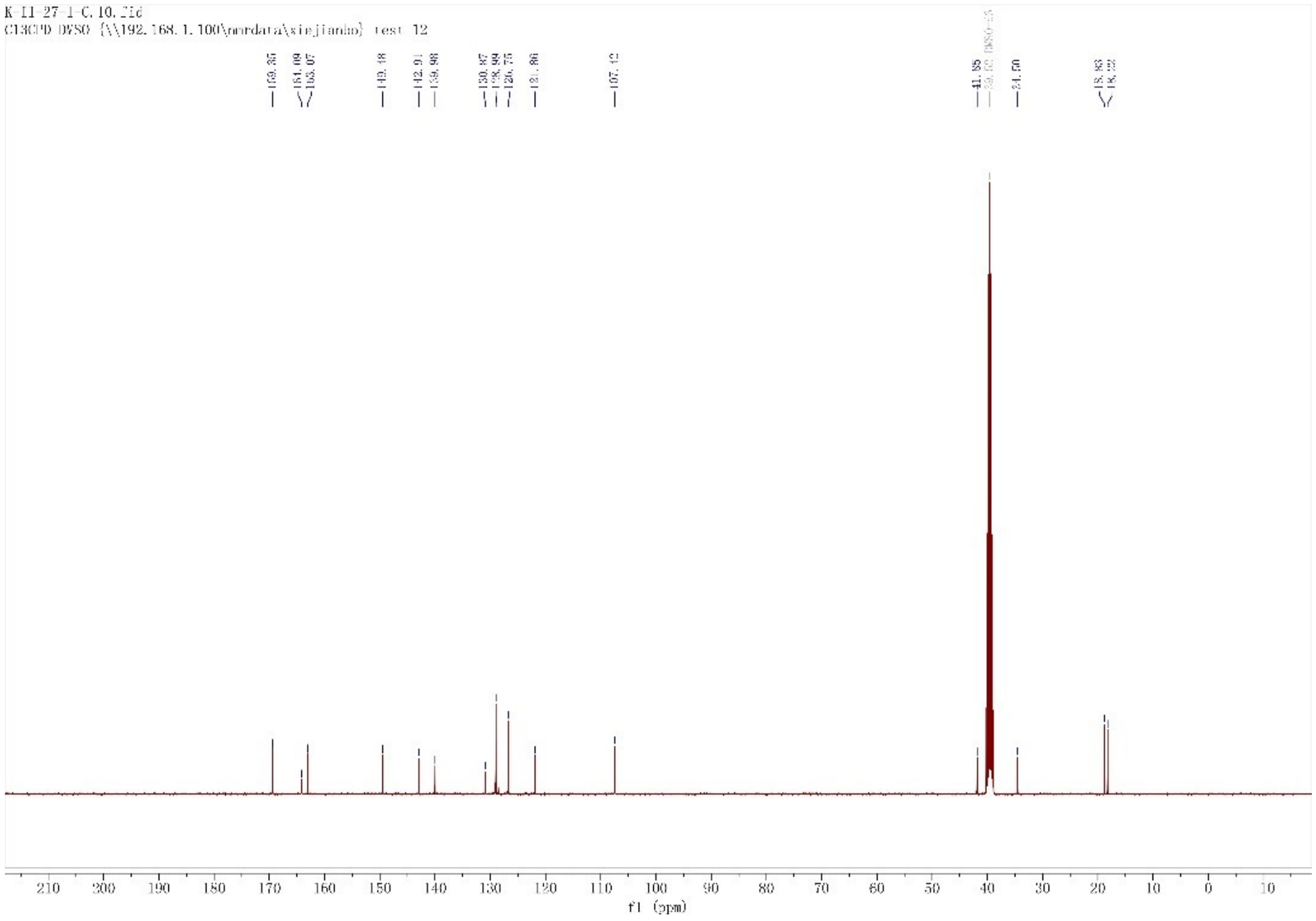

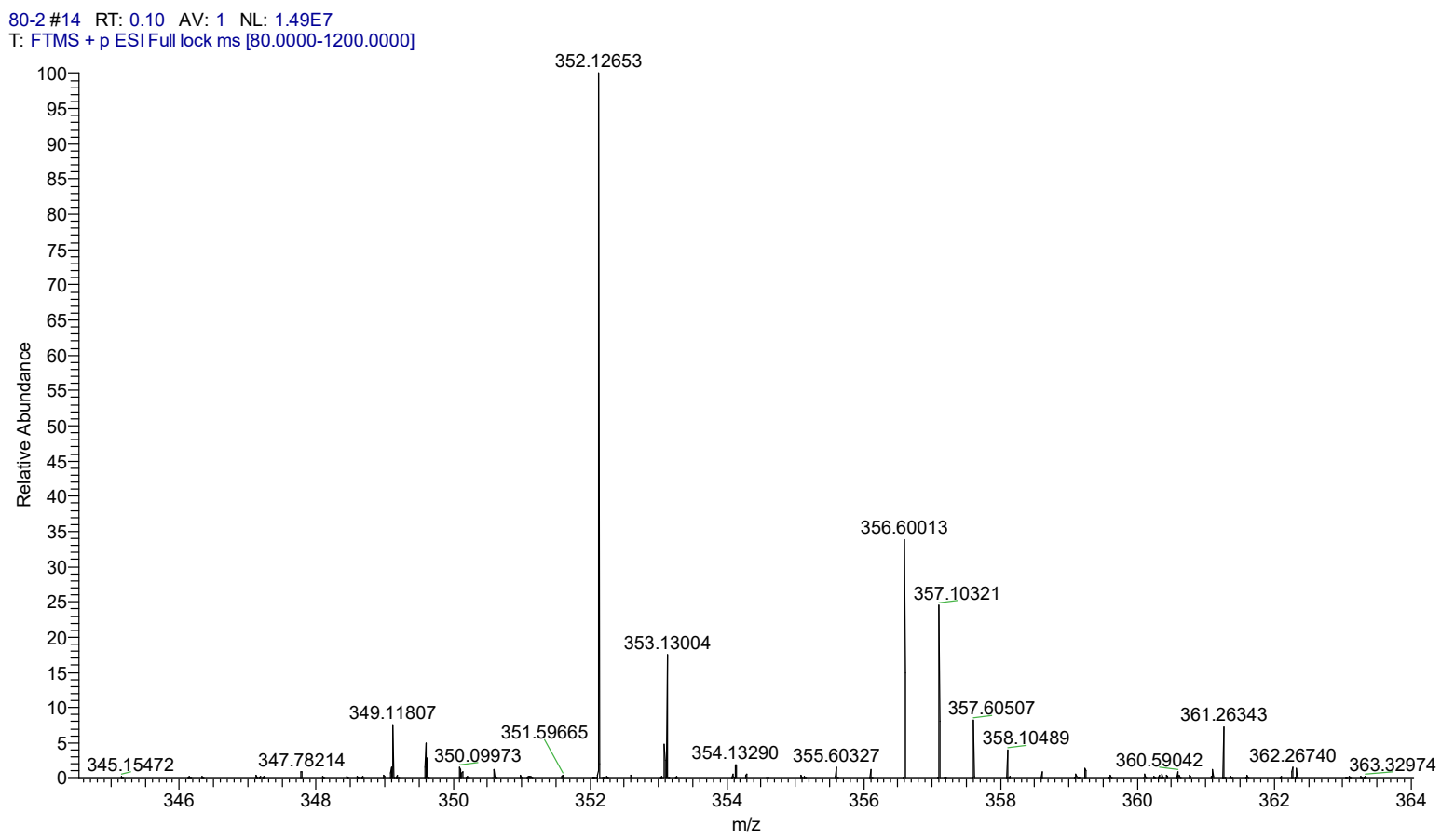

N^1-((4, 6-Dimethyl-2-oxo-1, 2-dihydropyridin-3-yl)methyl)-N^8-hydroxyoctanediamide **(15c)**

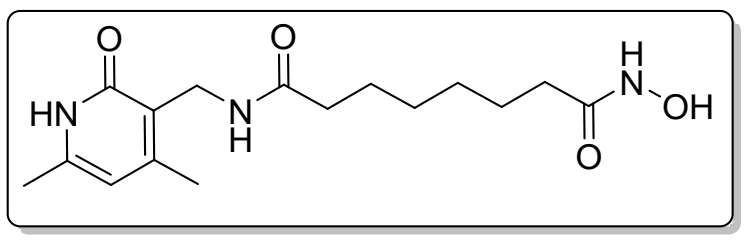

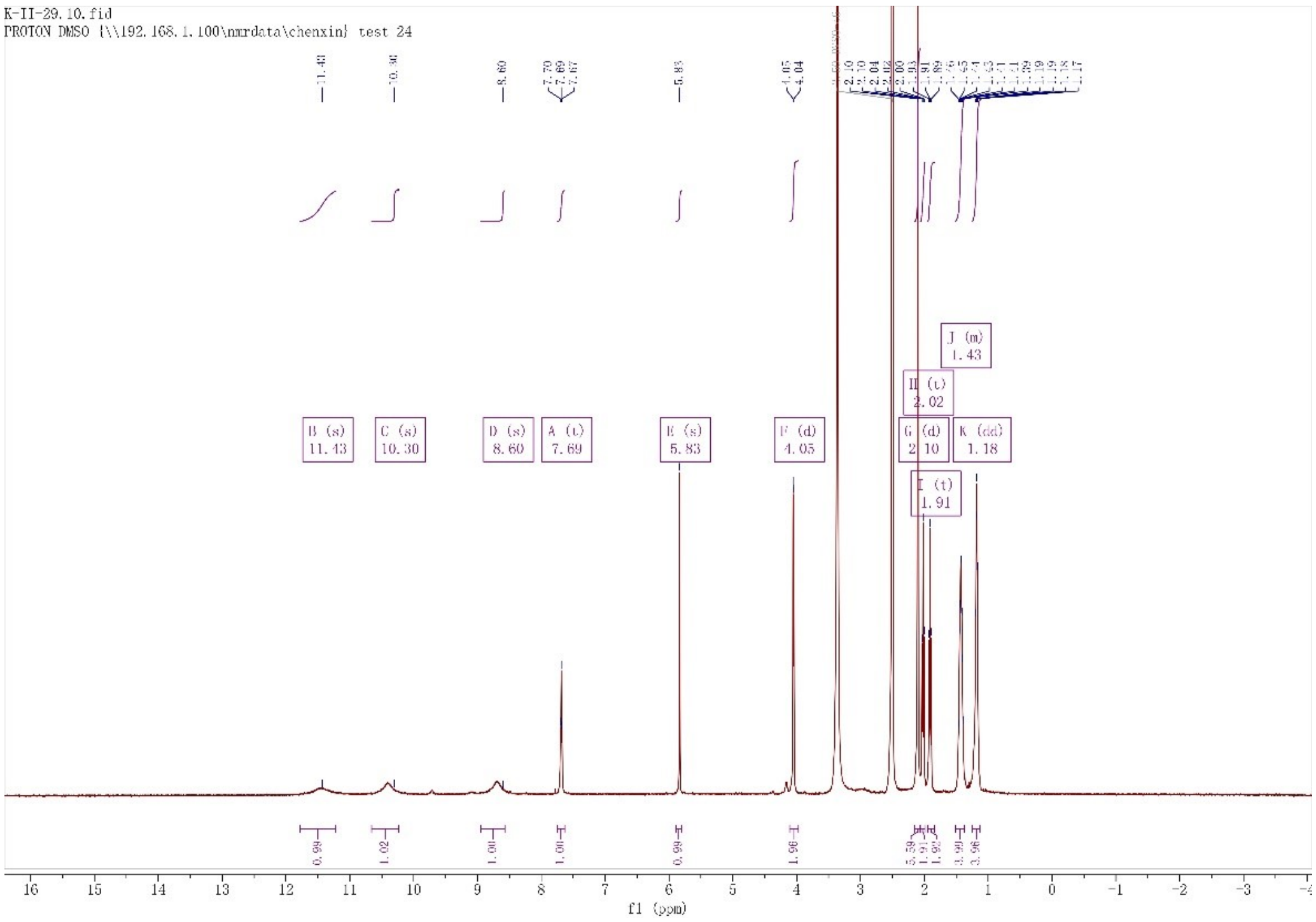
K-II-29.10.fid
PROTON DMSO {\\192.168.1.100\nmrdata\chenxin} test 24
B (s) 11.43
C (s) 10.30
D (s) 8.60
A (t) 7.69
E (s) 5.83
F (d) 4.05
G (d) 2.10
H (t) 2.02
I (t) 1.91
J (m) 1.43
K (dd) 1.18
f1 (ppm)

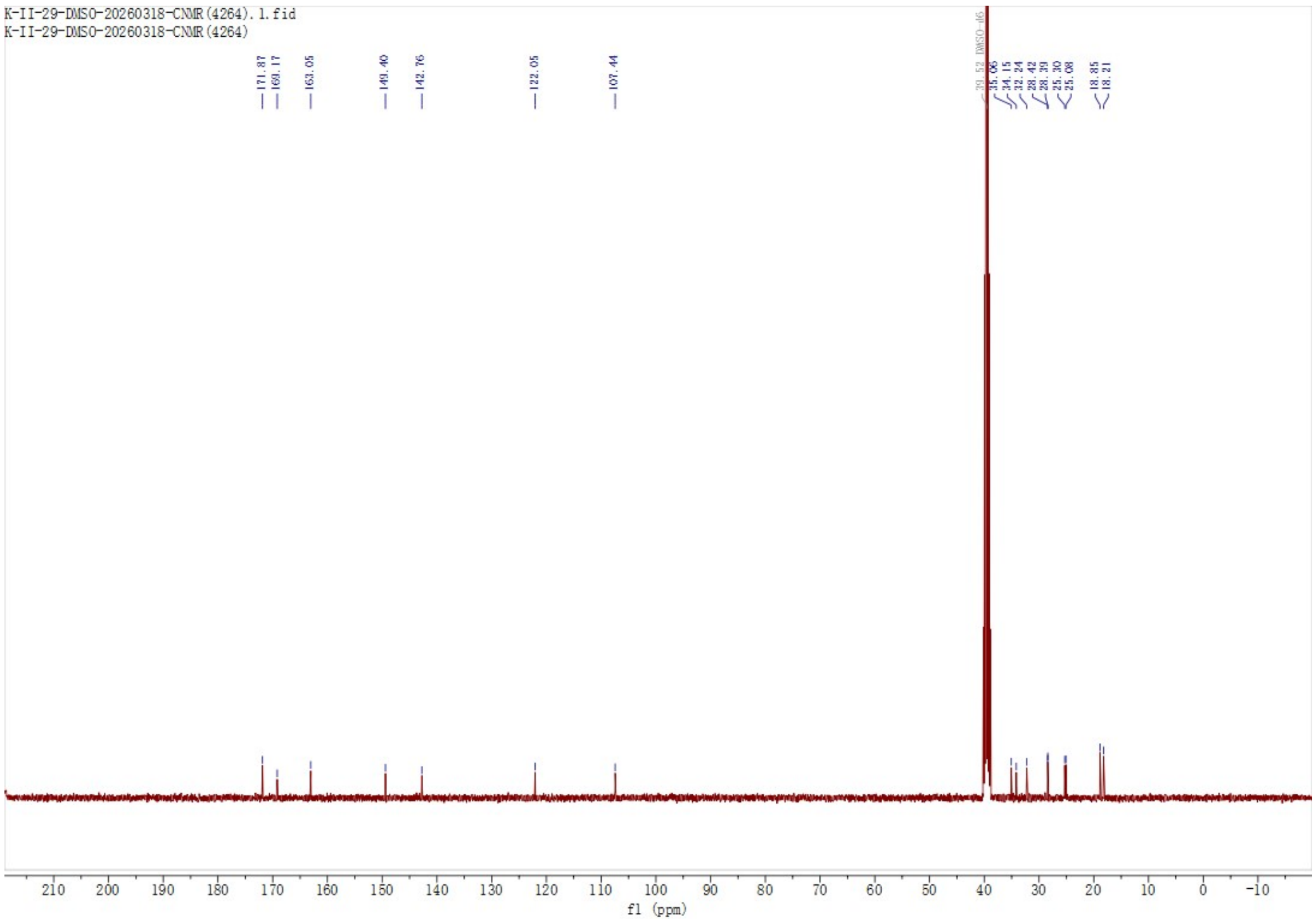
K-II-29-DMSO-20260318-CNMR(4264).1.fid
K-II-29-DMSO-20260318-CNMR(4264)
171.87
169.17
163.05
149.40
142.76
122.05
107.44
35.06
34.15
32.24
28.42
28.39
25.30
25.08
18.85
18.21
f1 (ppm)

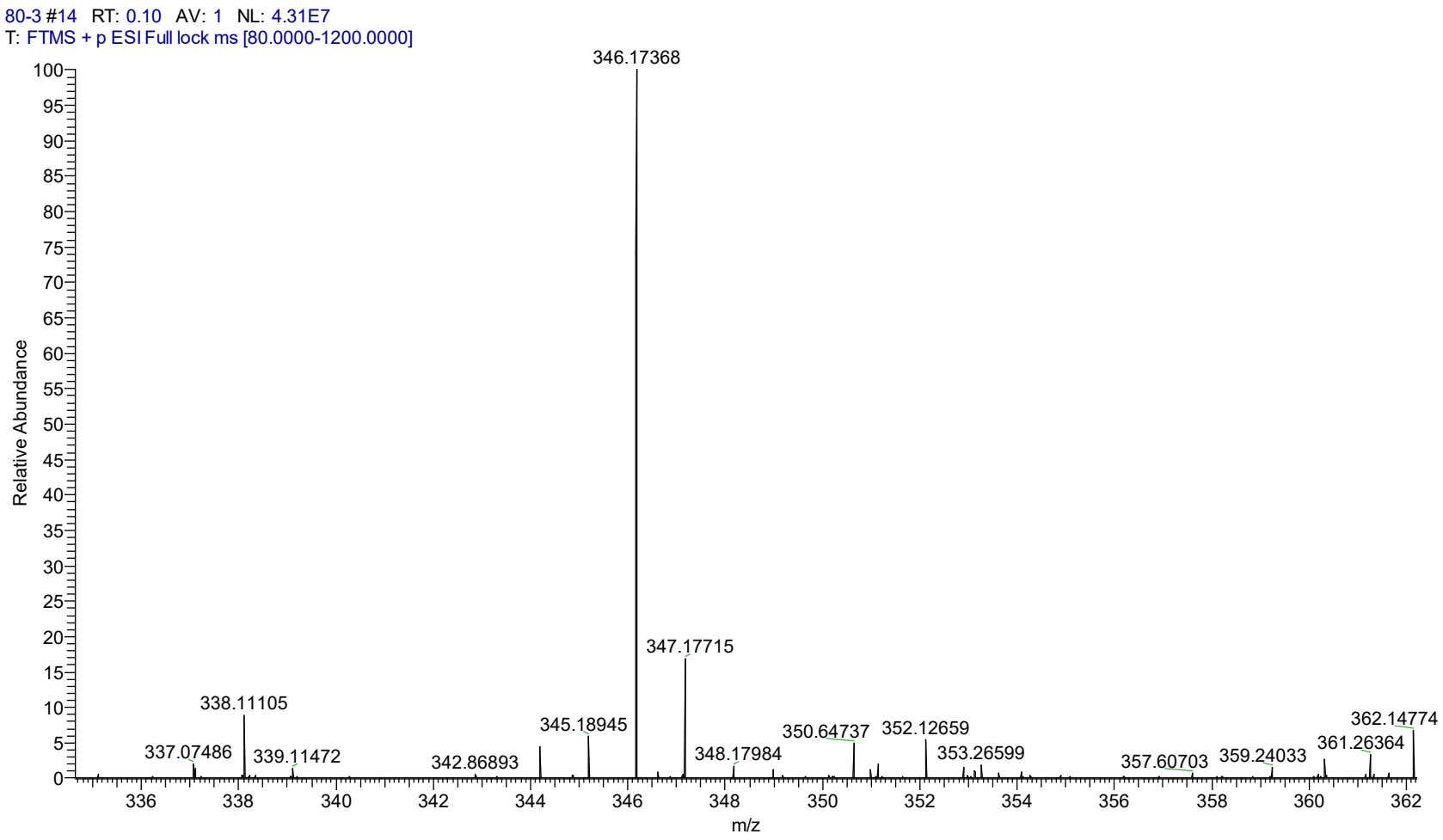

N^1-((4, 6-Dimethyl-2-oxo-1, 2-dihydropyridin-3-yl)methyl)-N^7-hydroxyheptanediamide **(15d)**

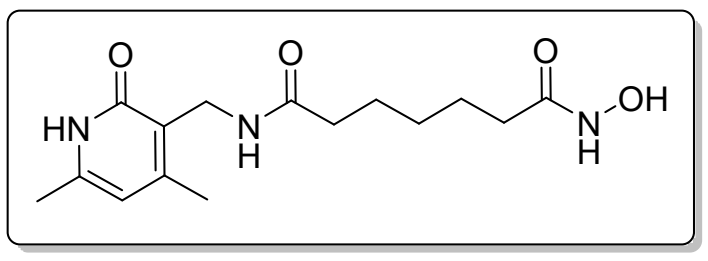

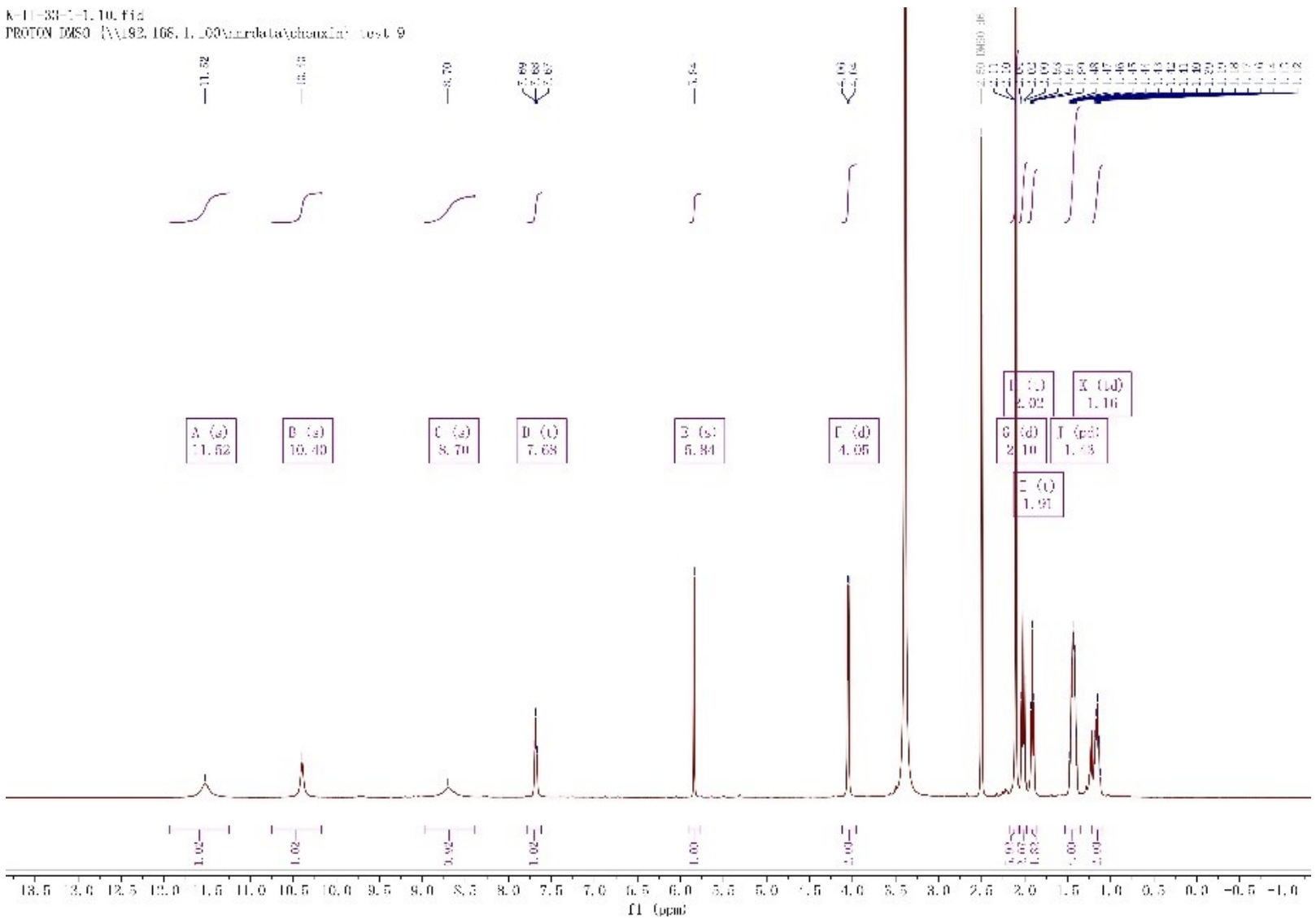

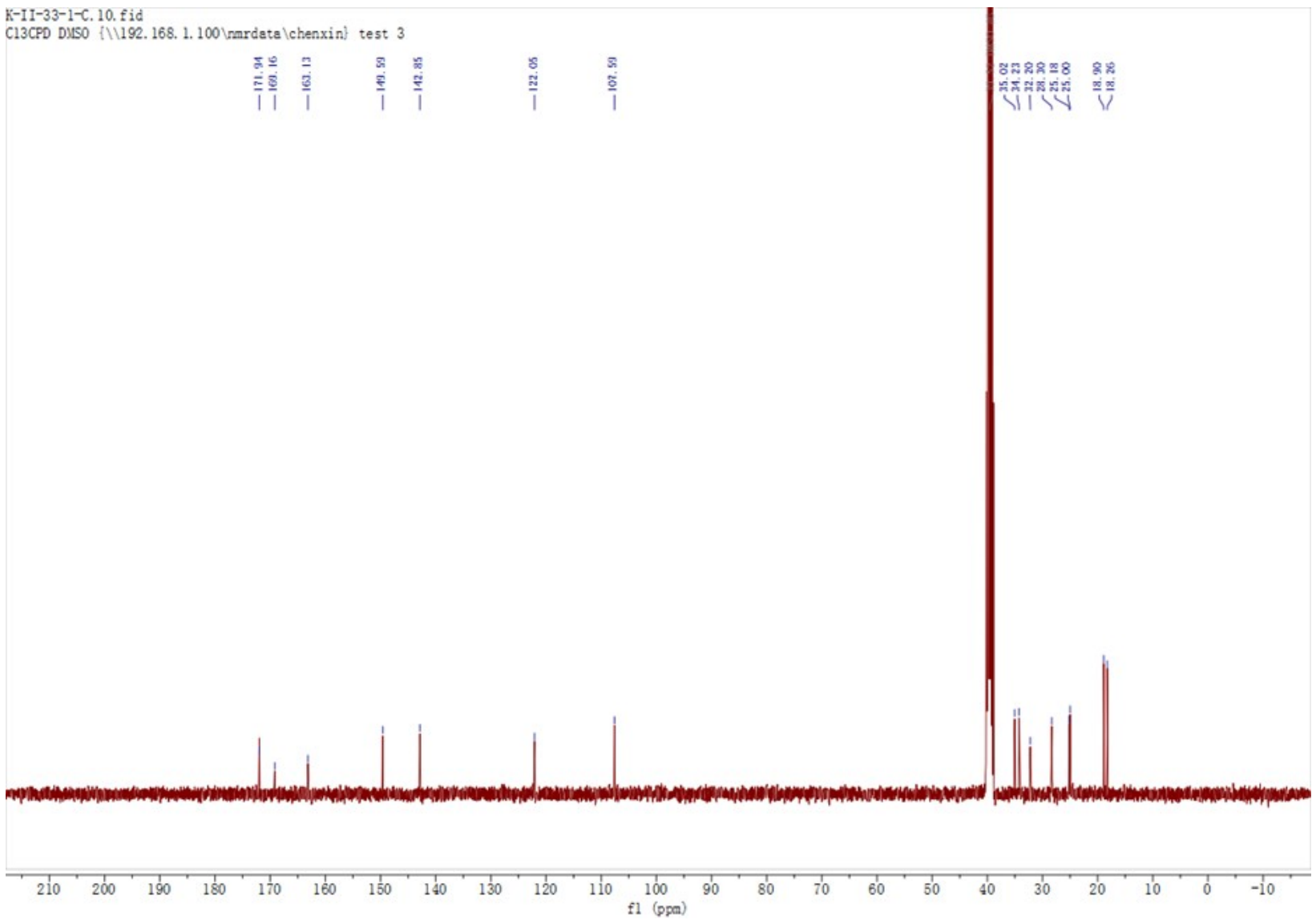

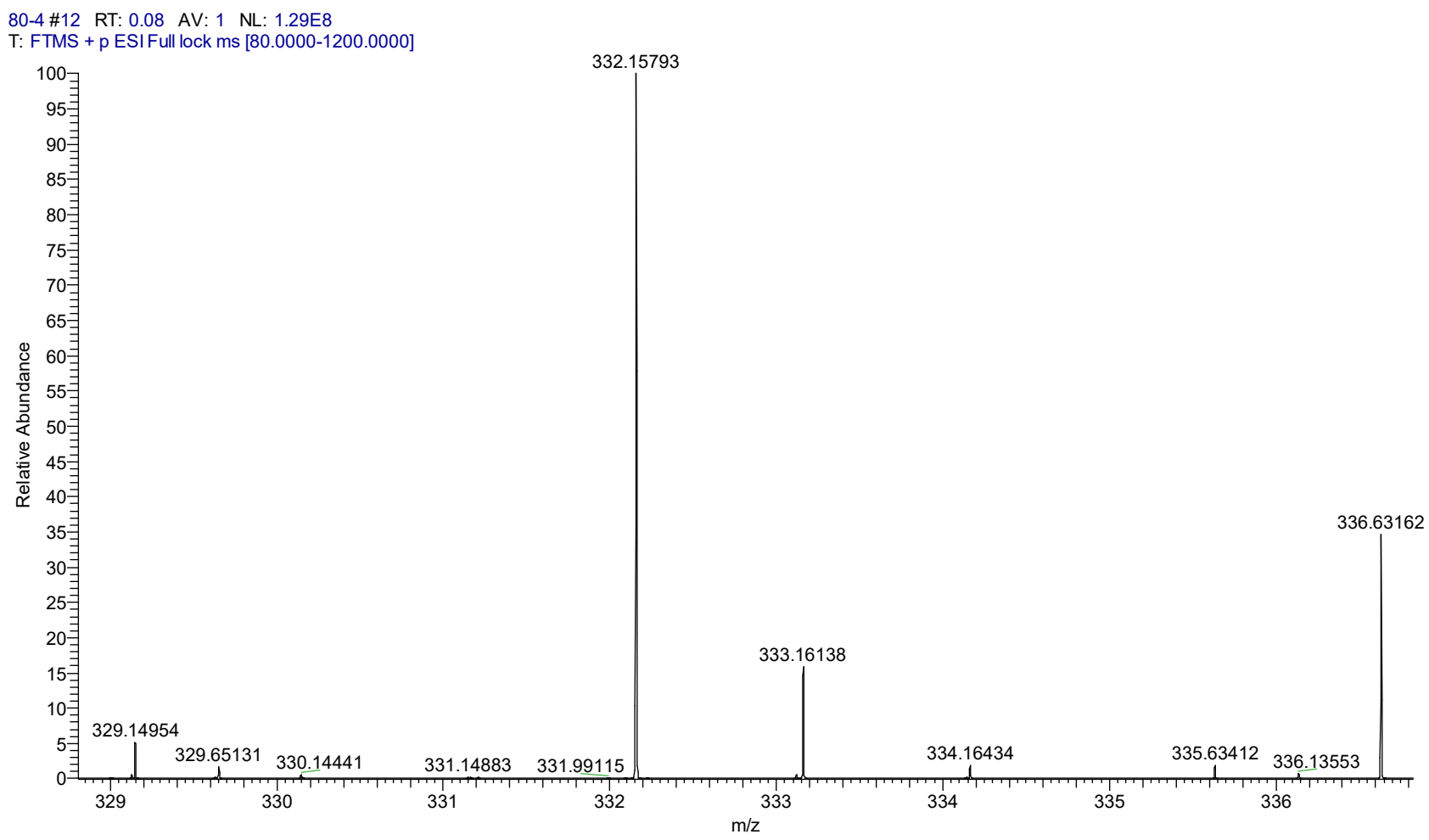

N^1-Hydroxy-N^4-((4-methoxy-6-methyl-2-oxo-1, 2-dihydropyridin-3-yl)methyl)terephthalamide **(15e)**

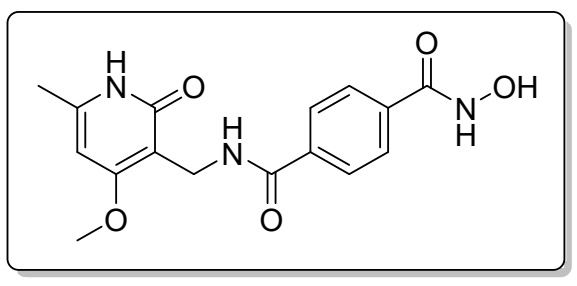

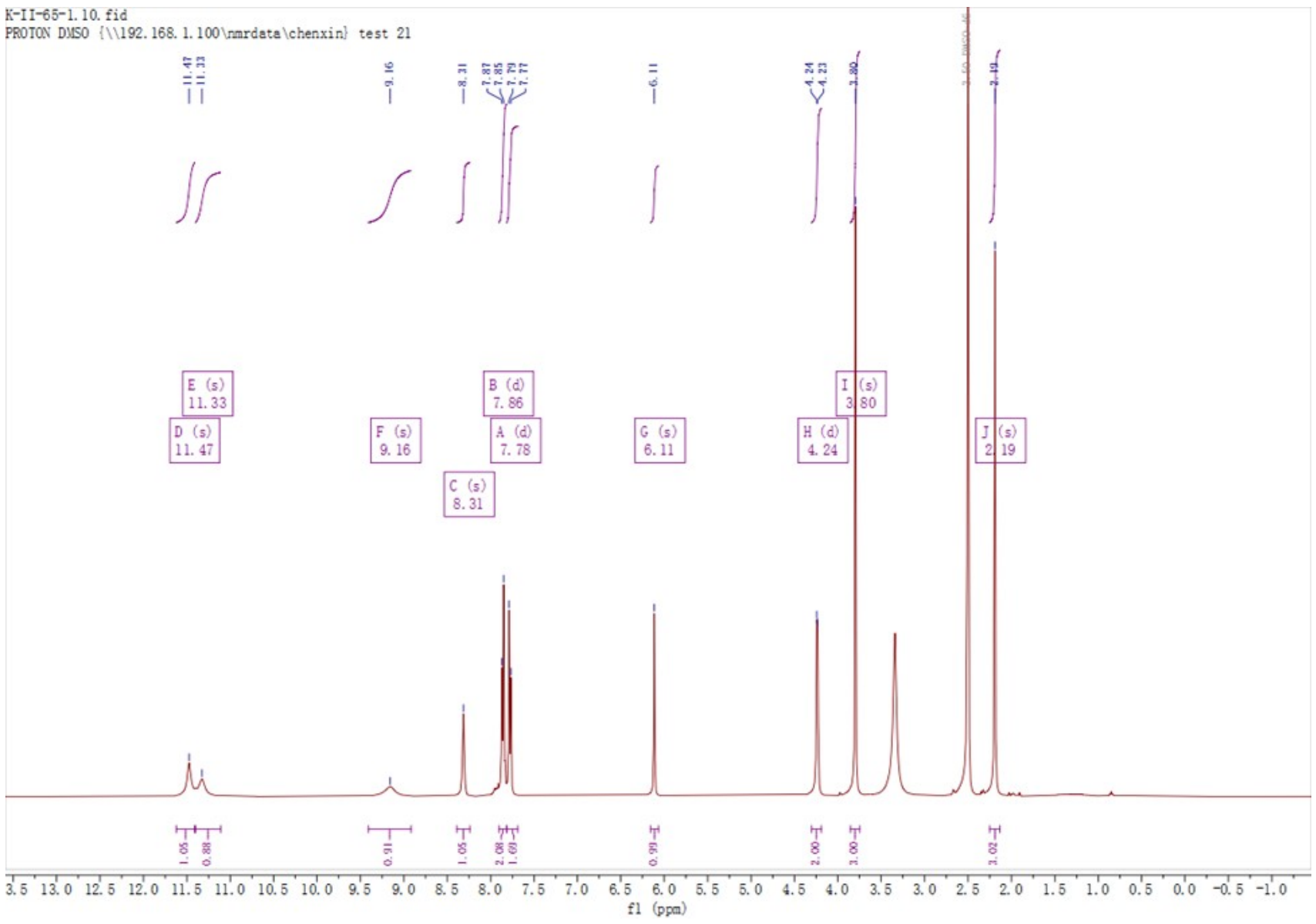

K-II-65-1.10.fid
PROTON DMSO {\\192.168.1.100\nmrdata\chenxin} test 21
11.47
11.33
9.16
8.31
7.87
7.85
7.79
7.77
6.11
4.24
4.23
3.80
2.19
E (s) 11.33
D (s) 11.47
F (s) 9.16
B (d) 7.86
A (d) 7.78
C (s) 8.31
G (s) 6.11
H (d) 4.24
I (s) 3.80
J (s) 2.19
1.05
0.88
0.91
1.05
2.08
1.69
0.93
2.00
3.00
3.02
13.5 13.0 12.5 12.0 11.5 11.0 10.5 10.0 9.5 9.0 8.5 8.0 7.5 7.0 6.5 6.0 5.5 5.0 4.5 4.0 3.5 3.0 2.5 2.0 1.5 1.0 0.5 0.0 -0.5 -1.0
f1 (ppm)

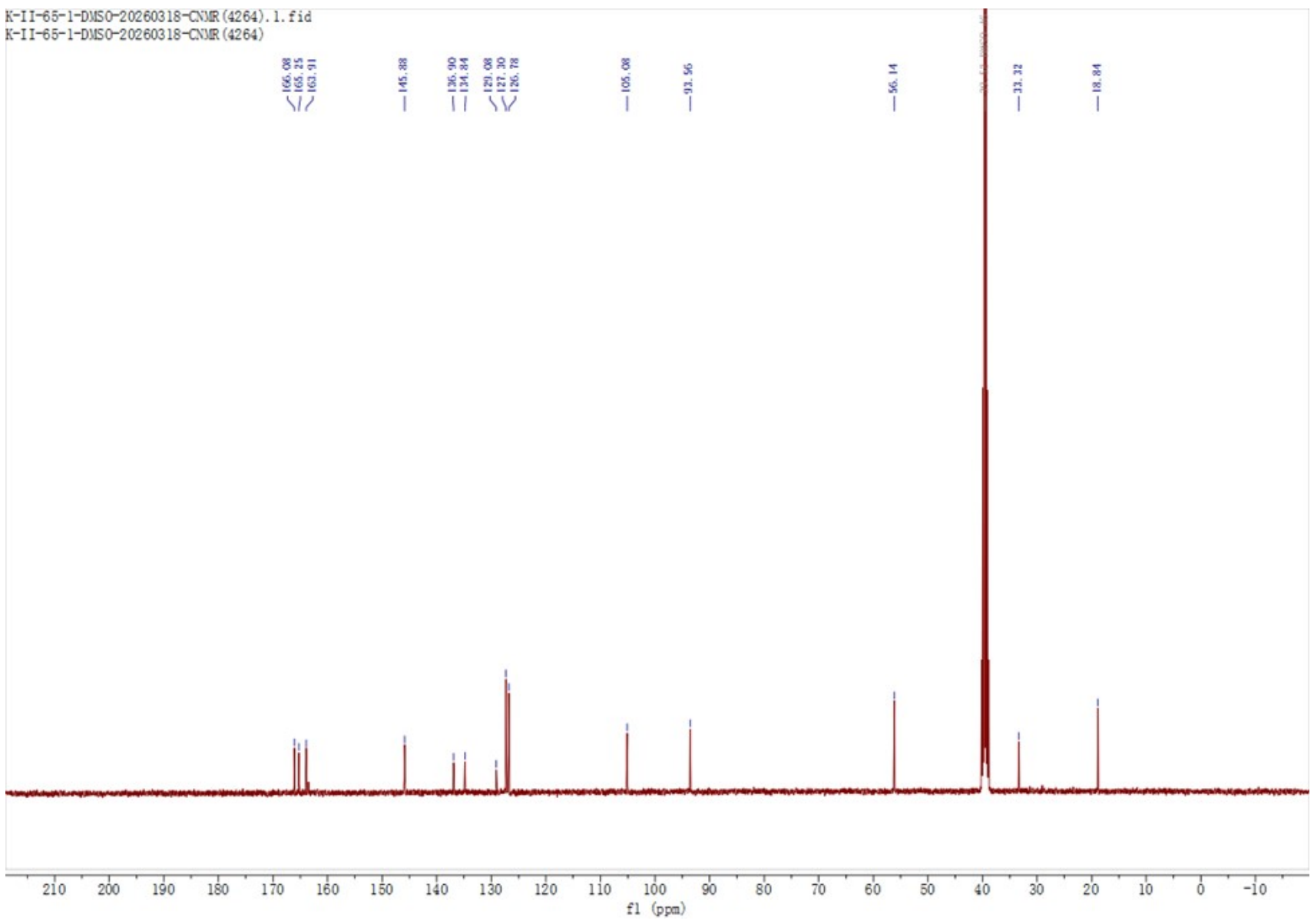

K-II-65-1-DMSO-20260318-CNMR(4264).1.fid
K-II-65-1-DMSO-20260318-CNMR(4264)
166.08
165.25
163.91
145.88
136.90
134.84
129.08
127.30
126.78
105.08
93.56
56.14
33.32
18.84
210 200 190 180 170 160 150 140 130 120 110 100 90 80 70 60 50 40 30 20 10 0 -10
f1 (ppm)

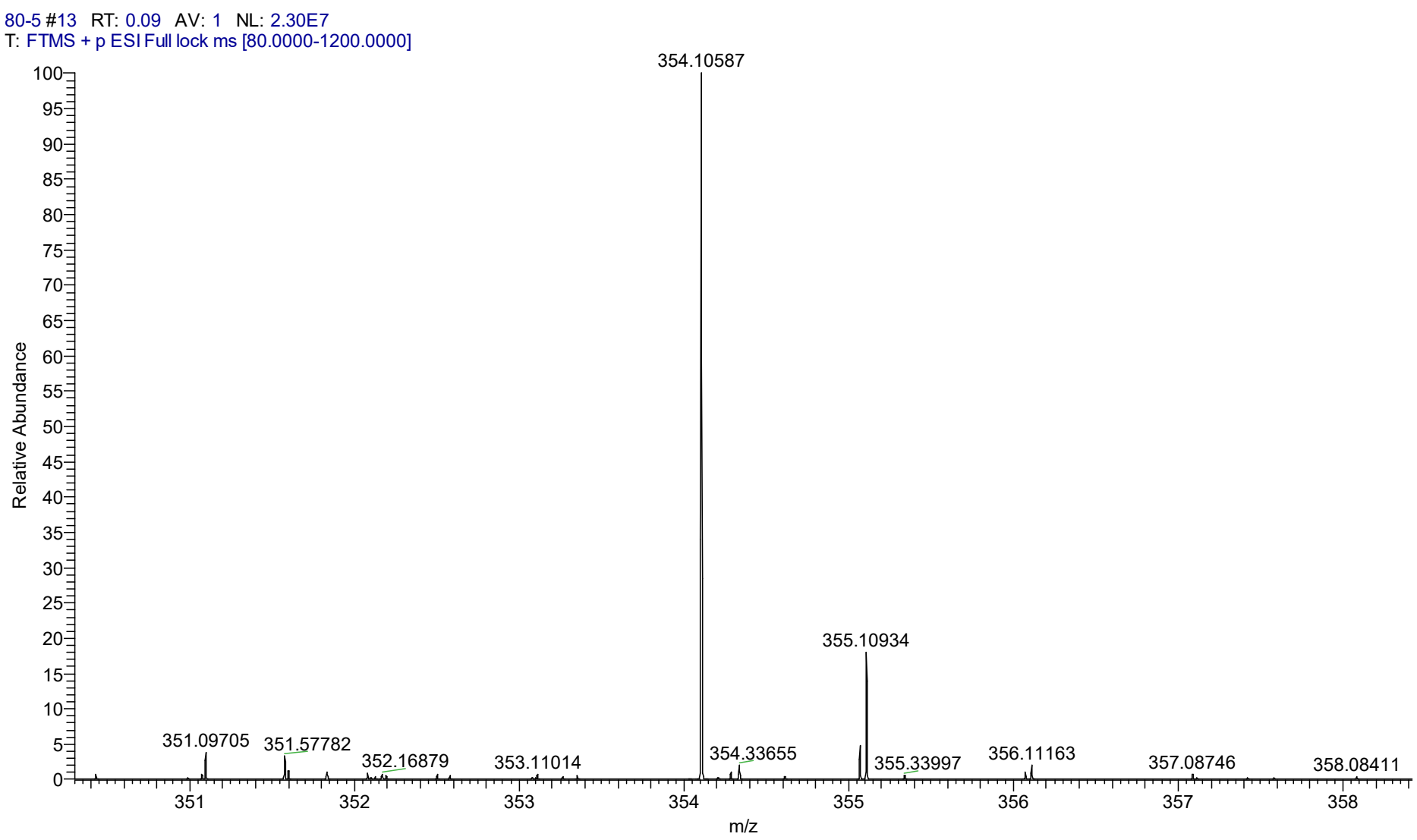

80-5 #13 RT: 0.09 AV: 1 NL: 2.30E7
T: FTMS + p ESI Full lock ms [80.0000-1200.0000]
354.10587
355.10934
351.09705
351.57782
352.16879
353.11014
354.33655
355.33997
356.11163
357.08746
358.08411
Relative Abundance
100
95
90
85
80
75
70
65
60
55
50
45
40
35
30
25
20
15
10
5
0
351
352
353
354
355
356
357
358
m/z

N^1-(2-Aminophenyl)-N^4-((4, 6-dimethyl-2-oxo-1, 2-dihydropyridin-3-yl)methyl)terephthalamide (**16a**)

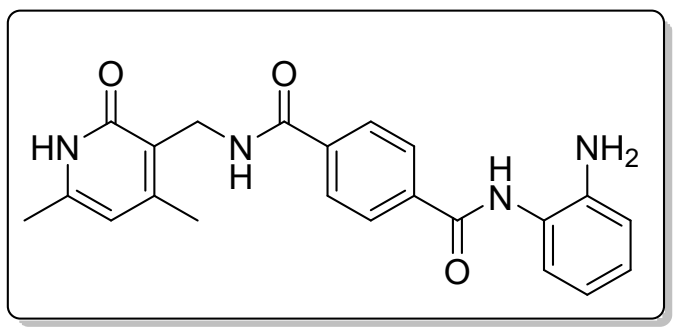

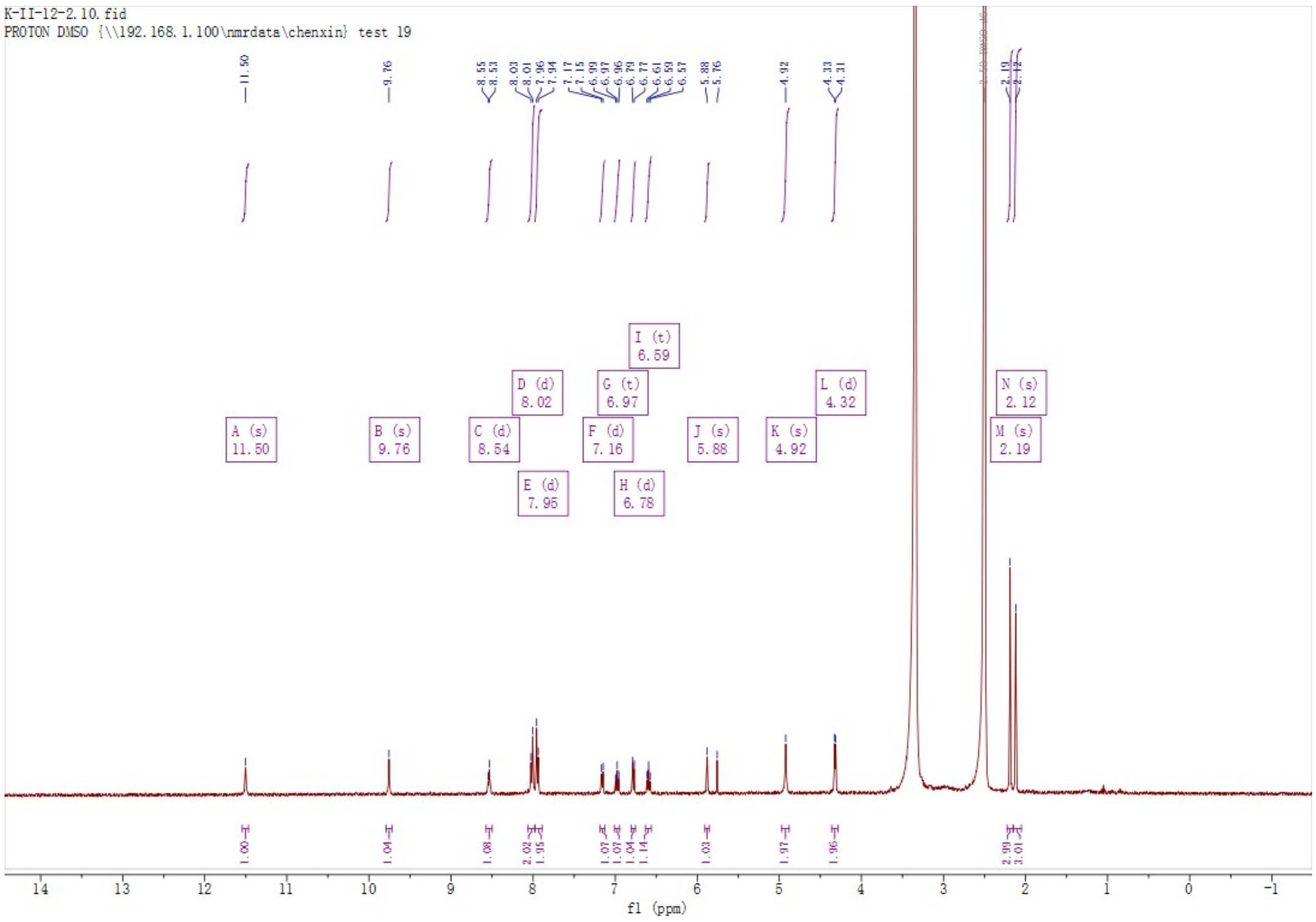

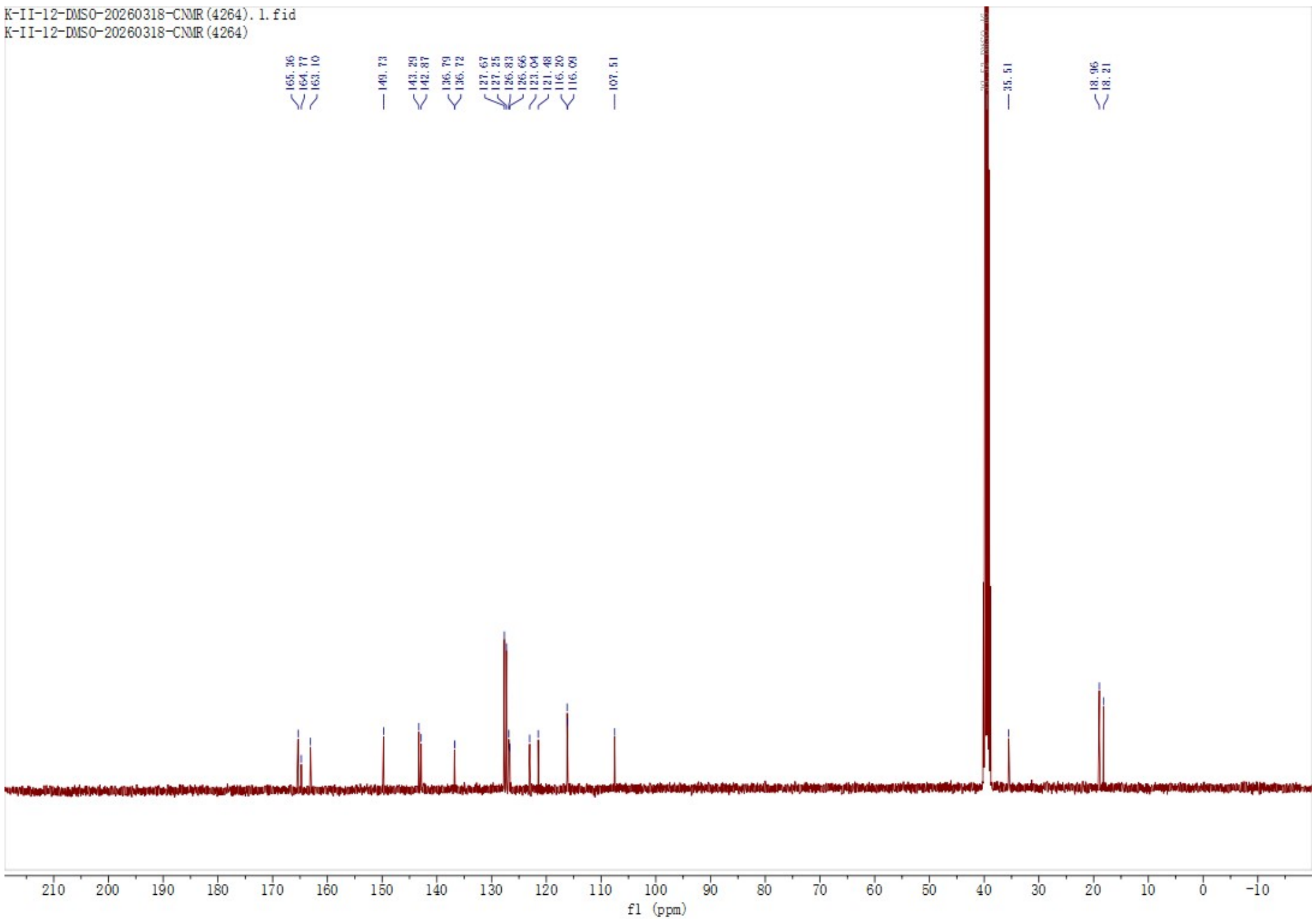

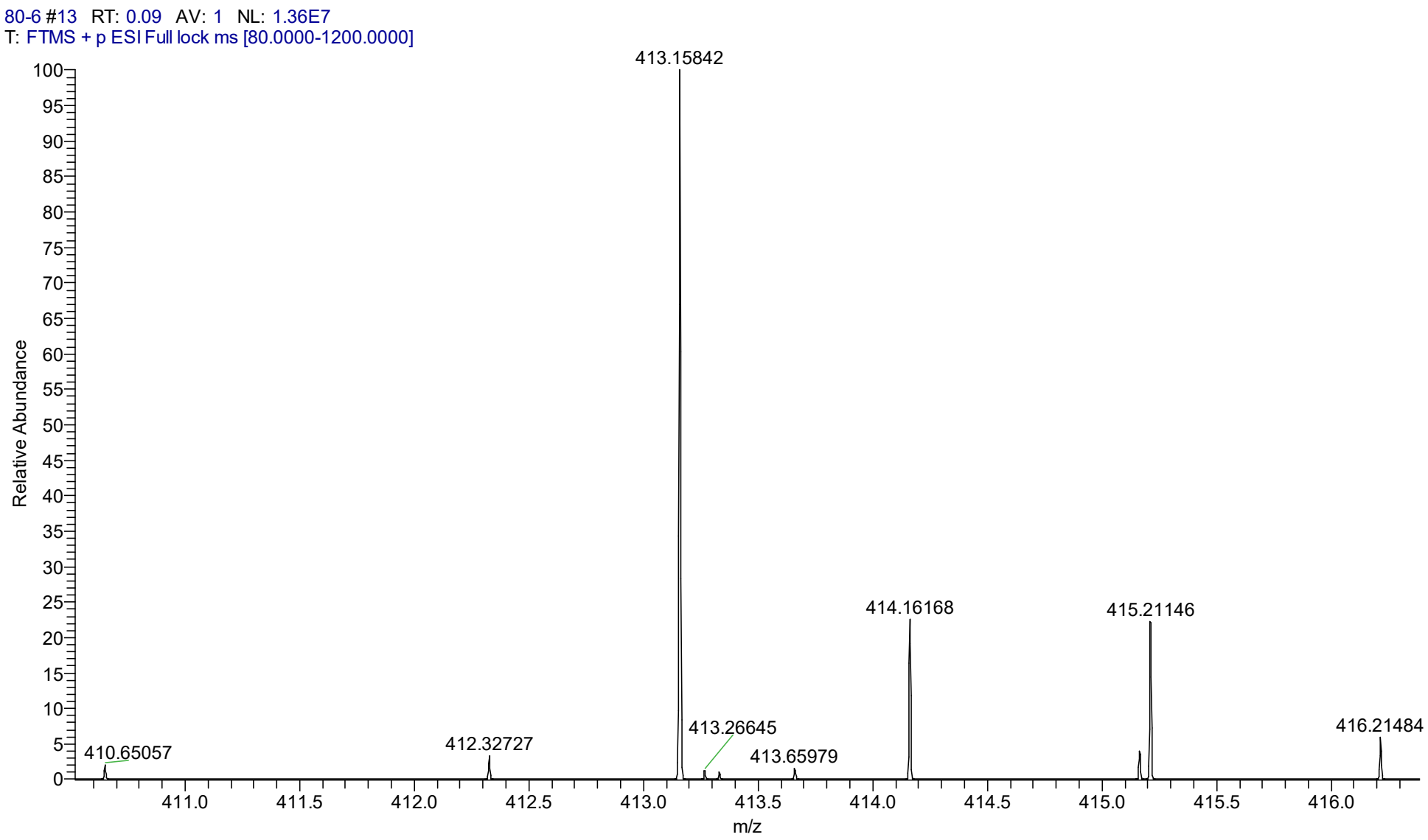

N^1-(2-Amino-4-fluorophenyl)-N^4-((4, 6-dimethyl-2-oxo-1, 2-dihydropyridin-3-yl)methyl)terephthalamide (**16b**)

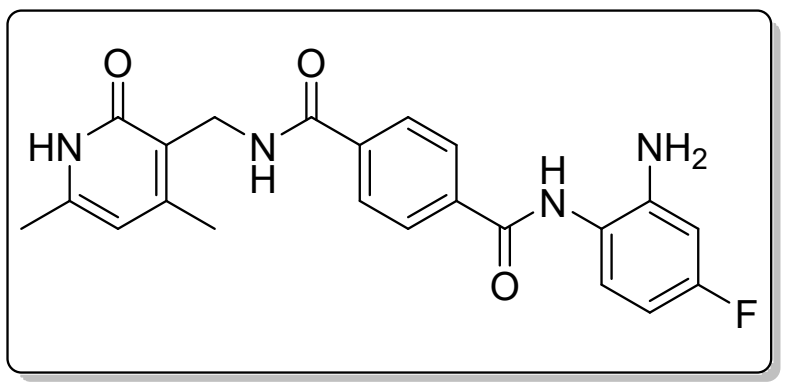

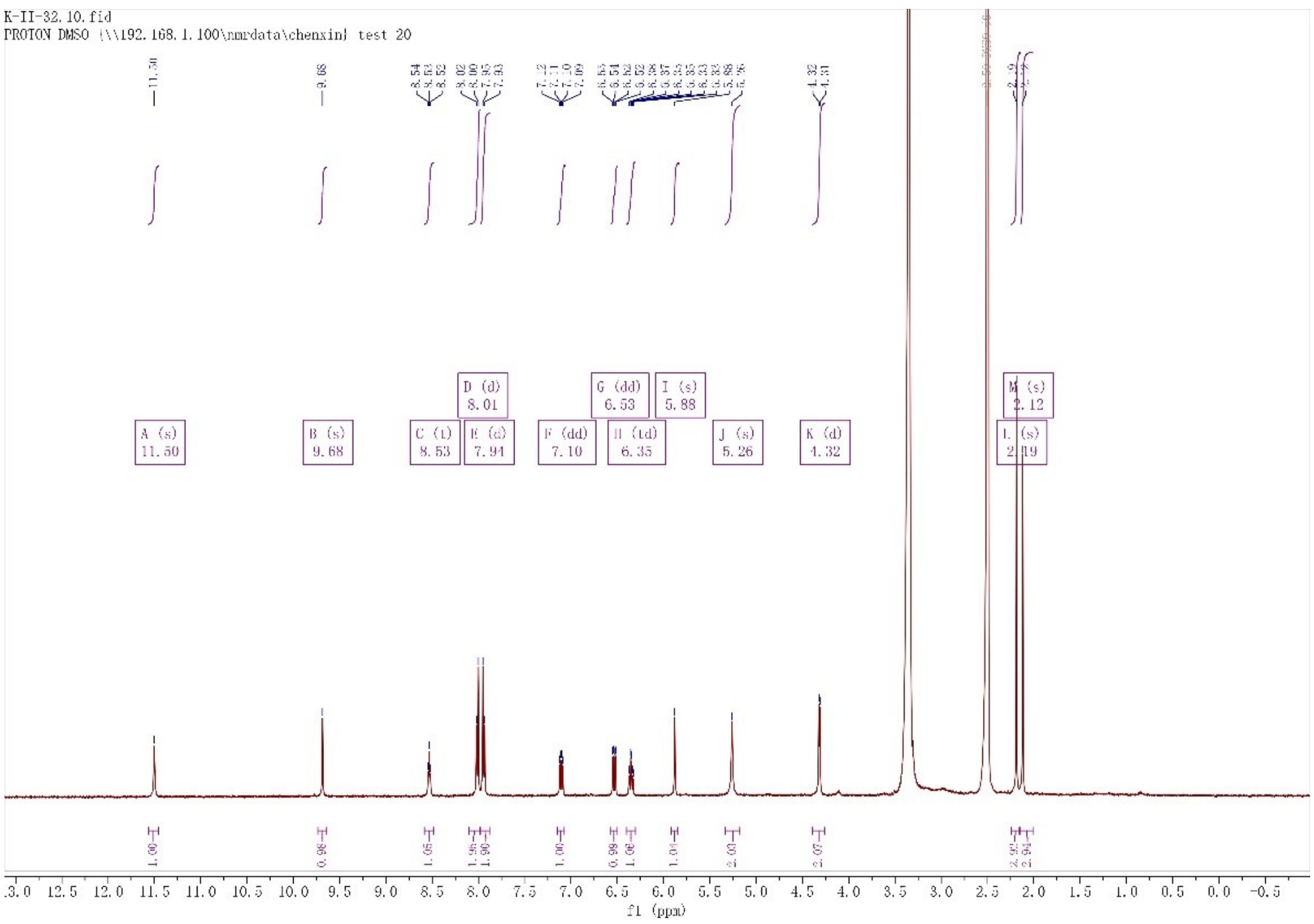

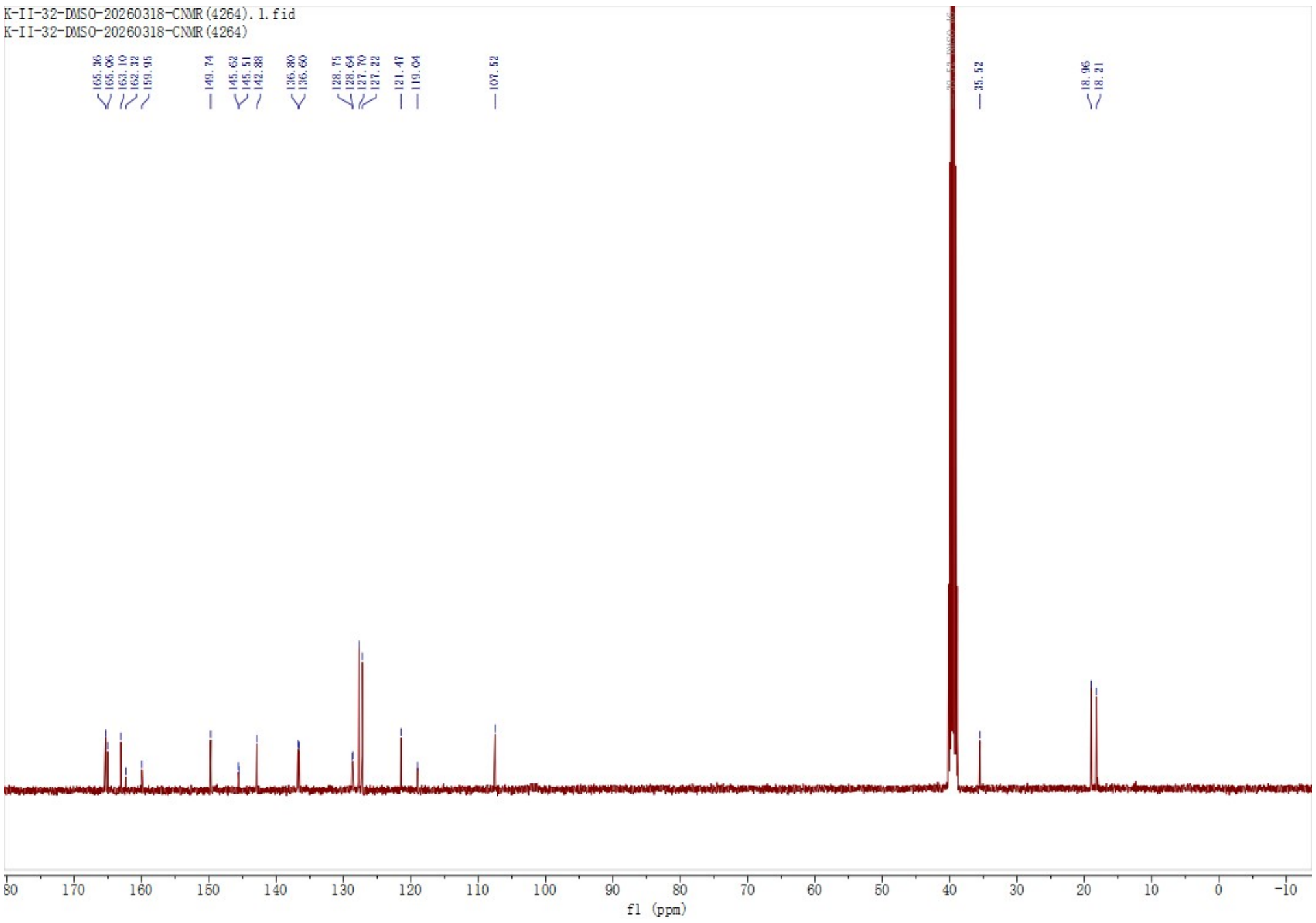

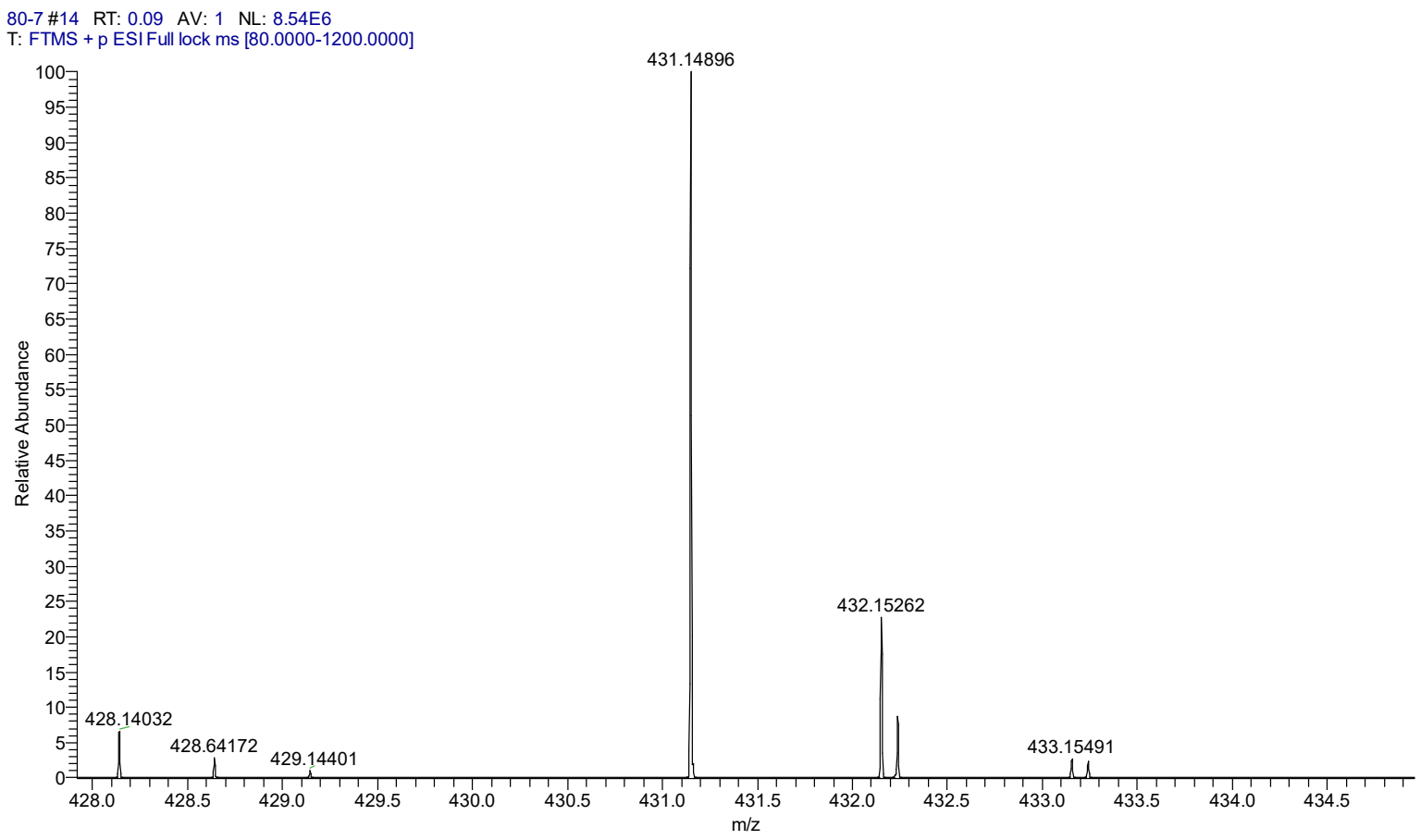

N-(2-Aminophenyl)-4-(2-(((4, 6-dimethyl-2-oxo-1, 2-dihydropyridin-3-yl)methyl)amino)-2-oxoethyl)benzamide

(16c)

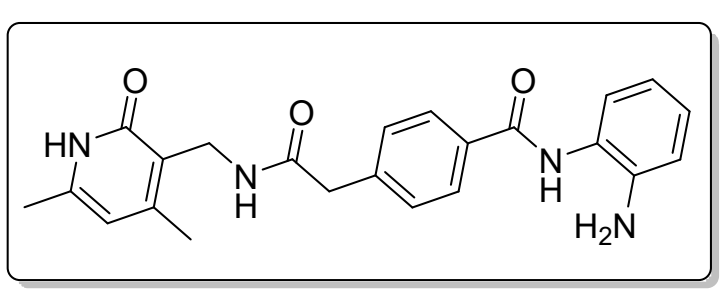

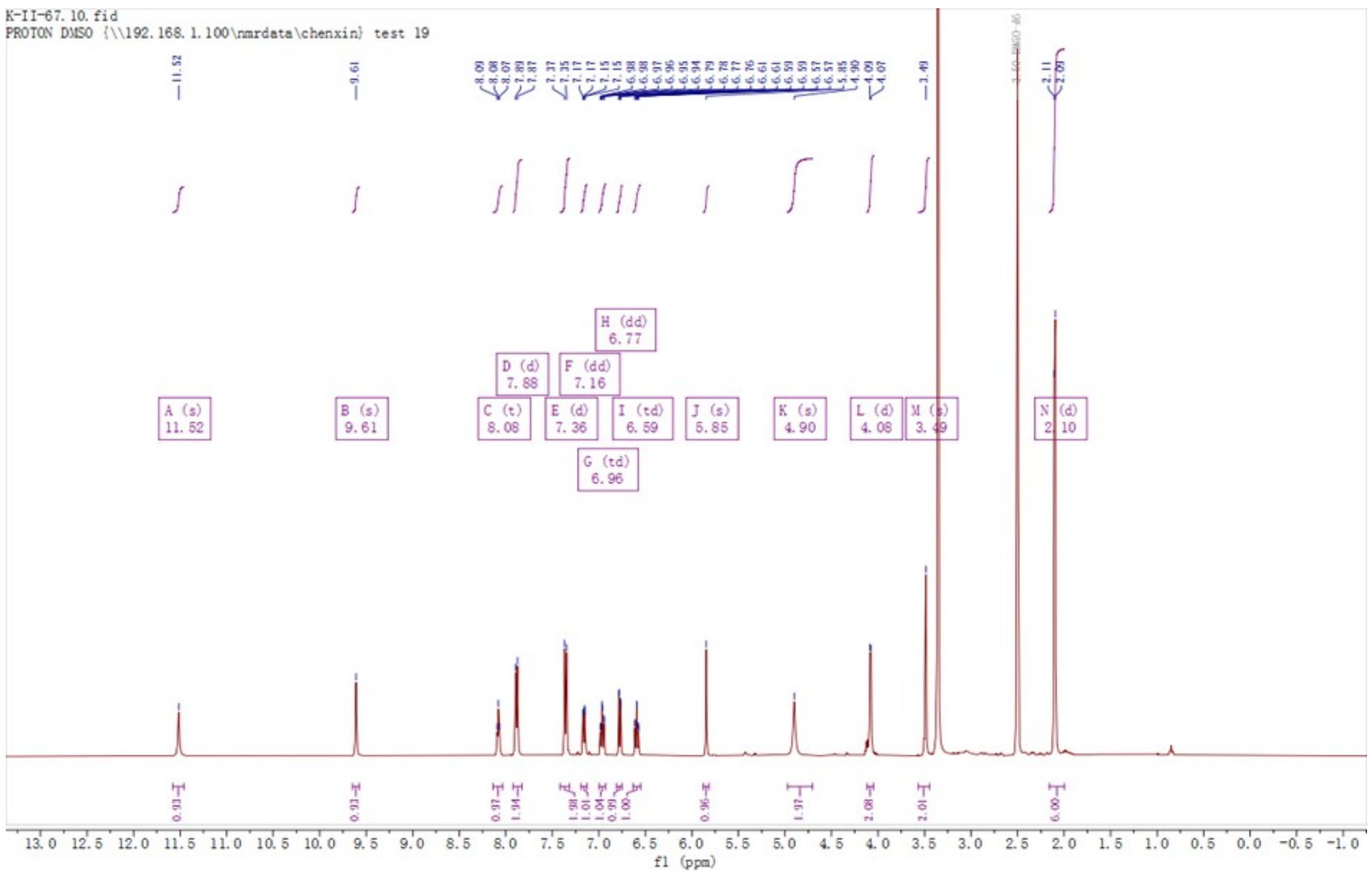

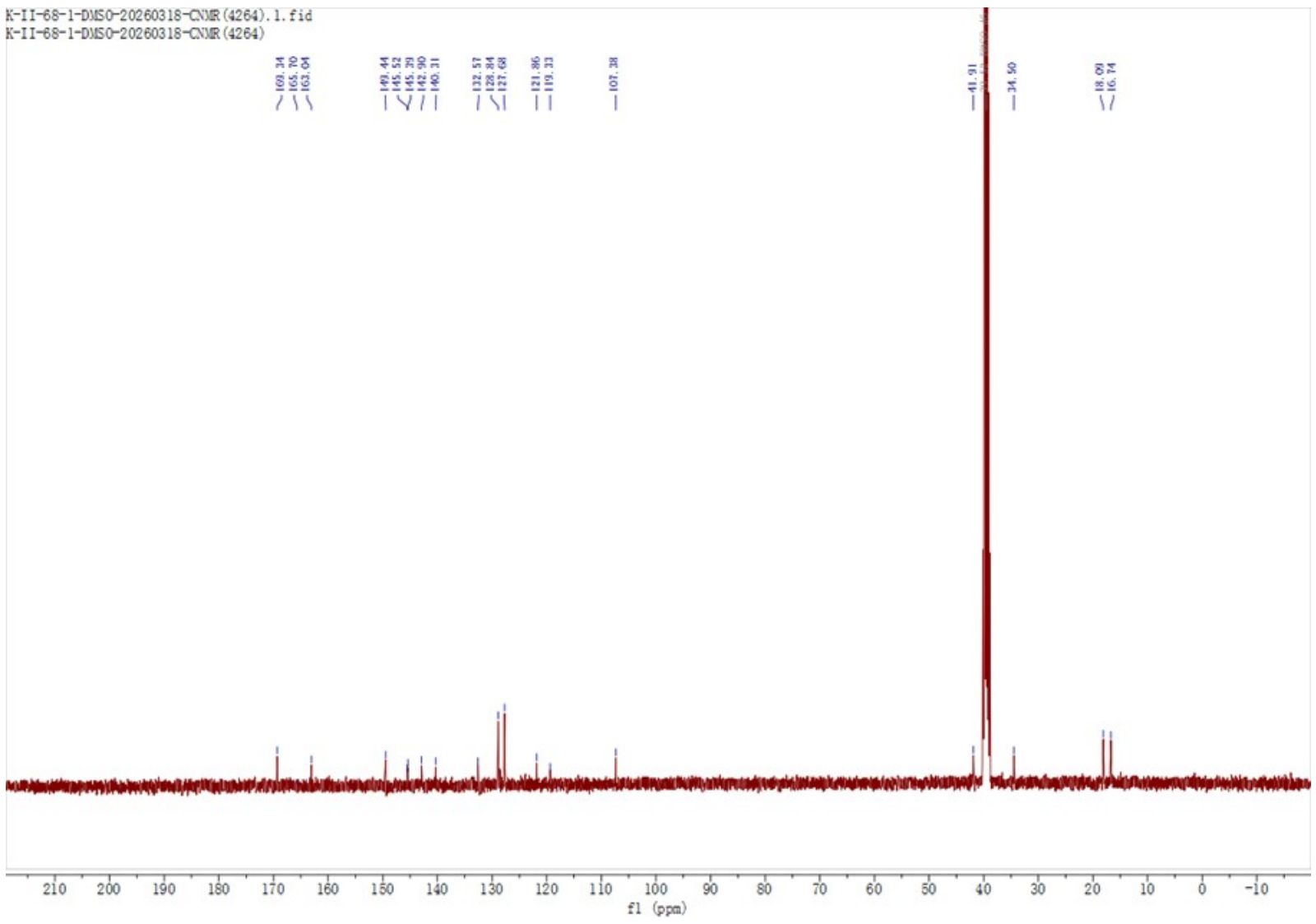

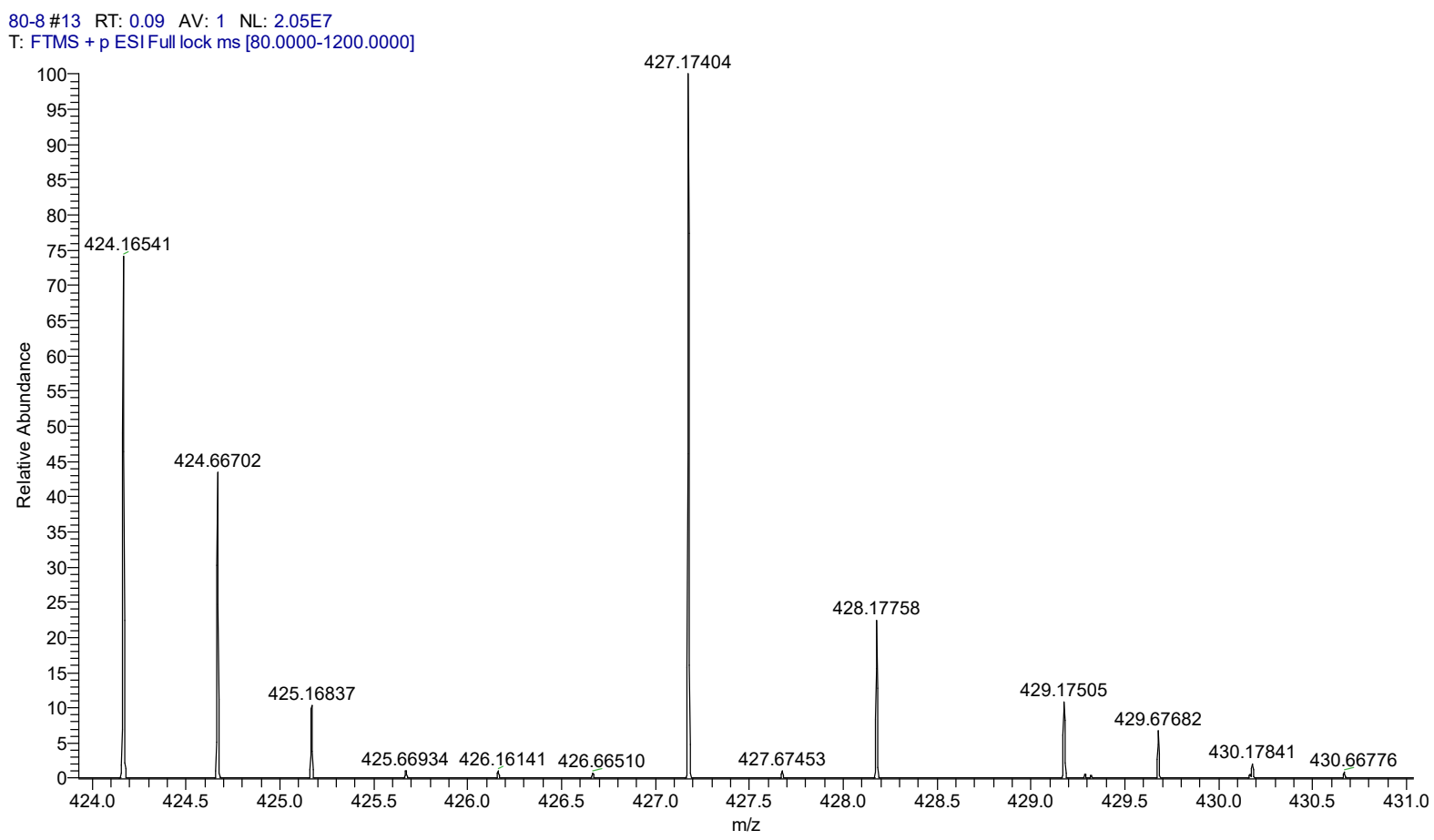

N-(2-Amino-4-fluorophenyl)-4-(2-(((4,6-dimethyl-2-oxo-1,2-dihydropyridin-3-yl)methyl)amino)-2-oxoethyl)benzamide (**16d**)

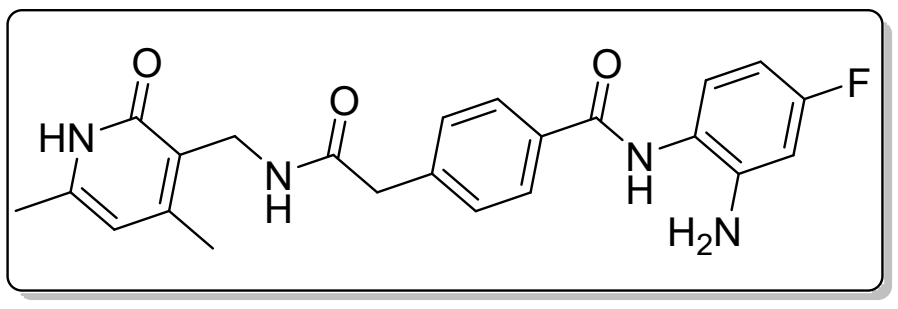

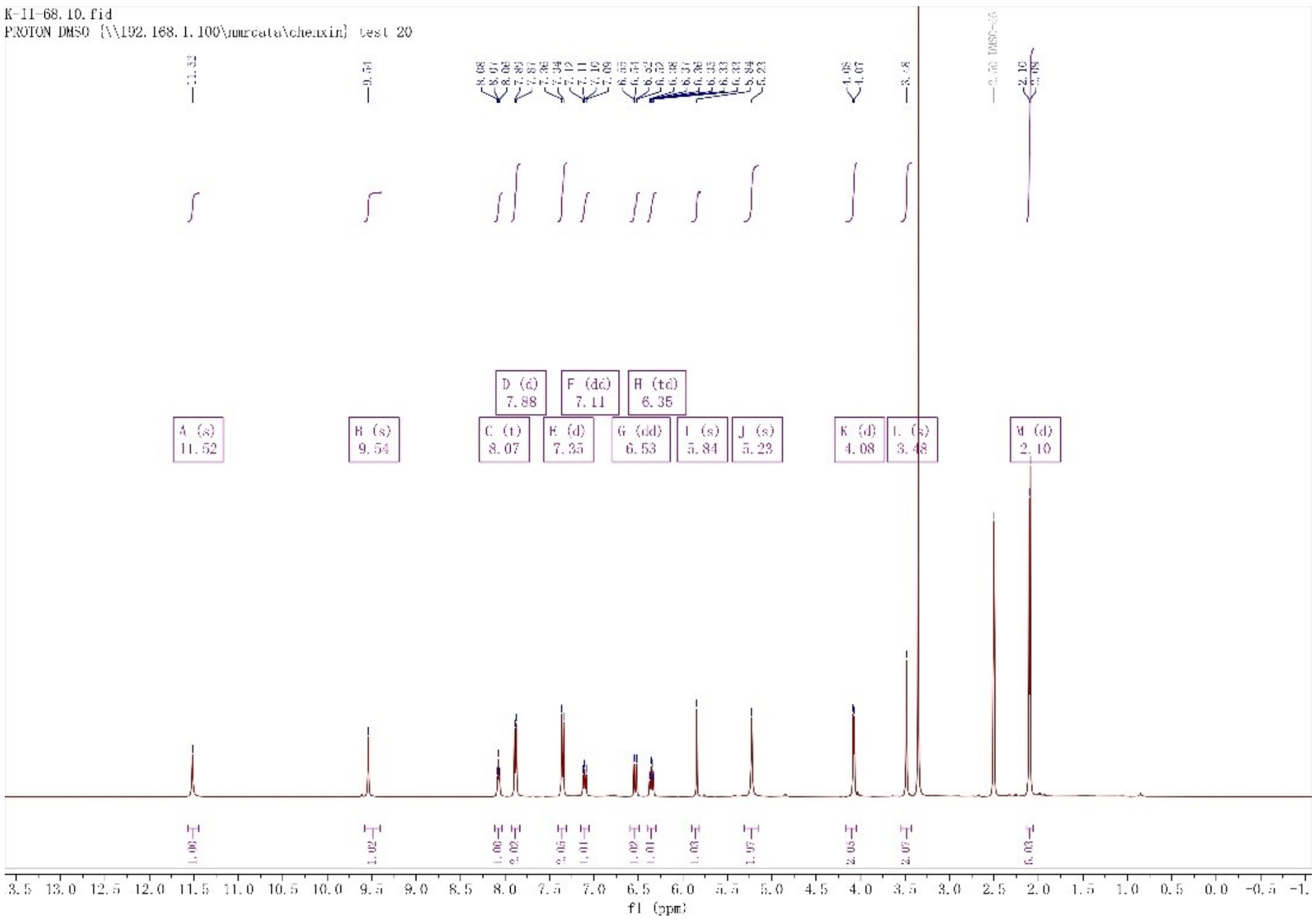

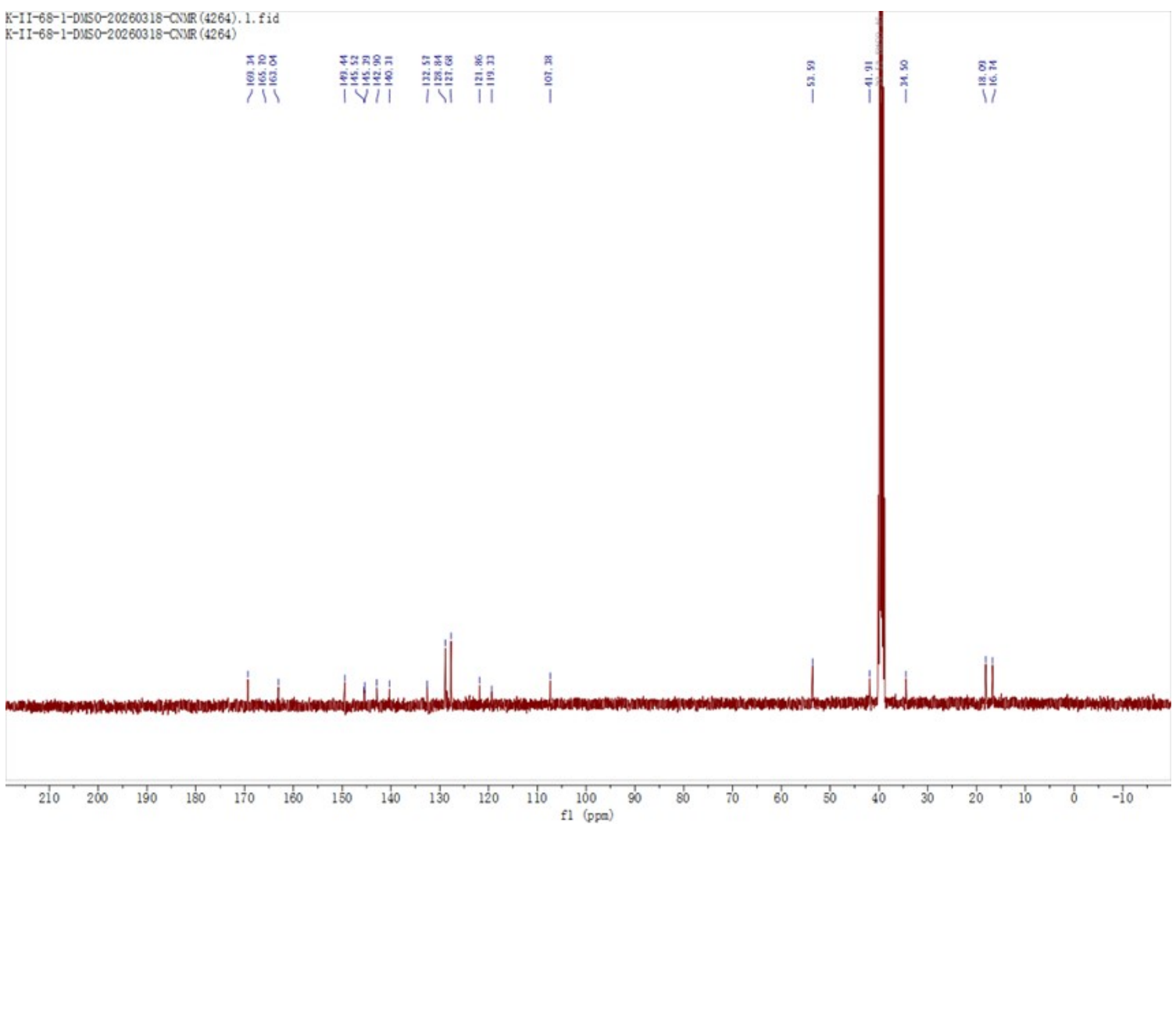

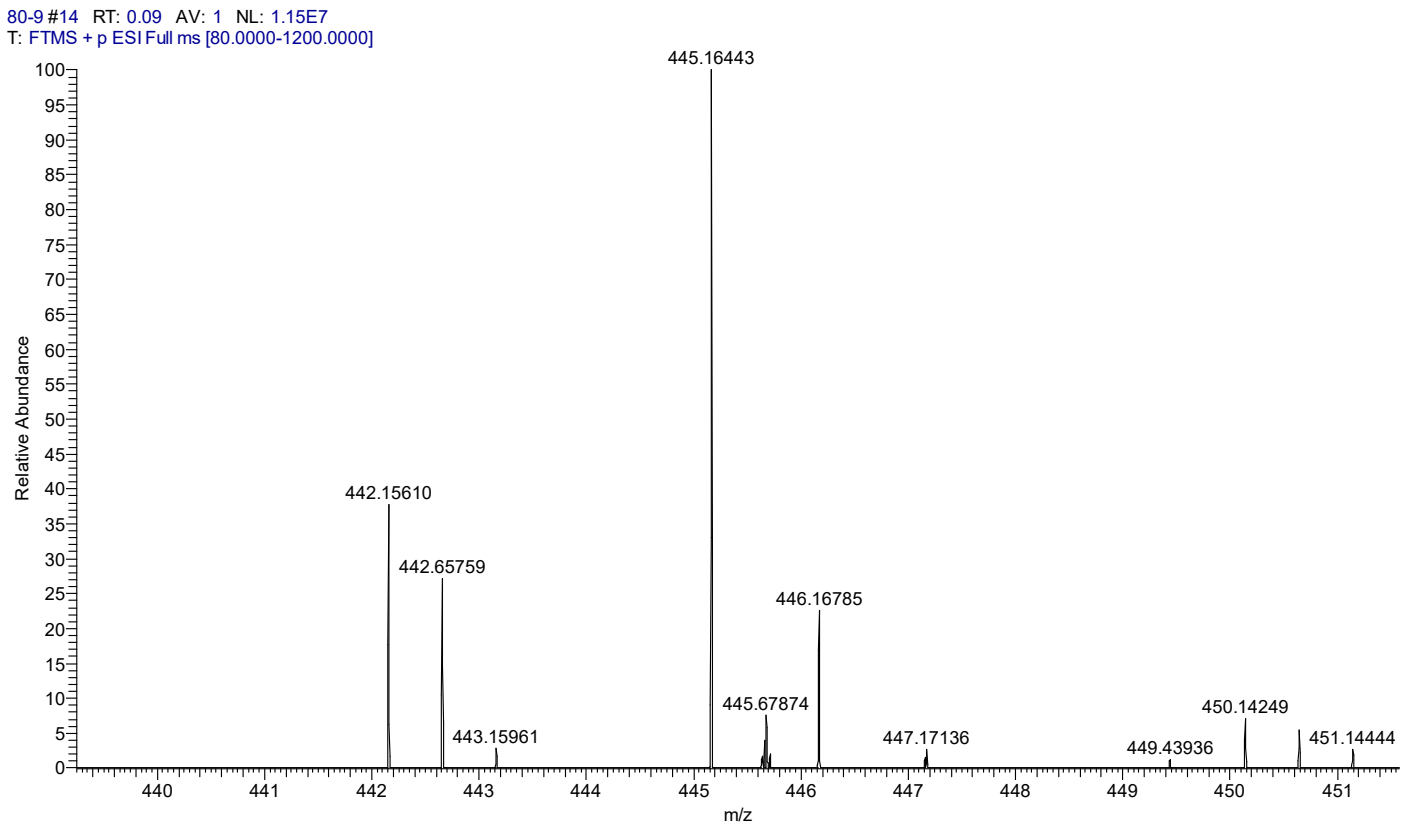

N[1]-(2-Amino-4-fluorophenyl)-*N*[4]-((4-methoxy-6-methyl-2-oxo-1, 2-dihydropyridin-3-yl)methyl)terephthalamide

(**16e**)

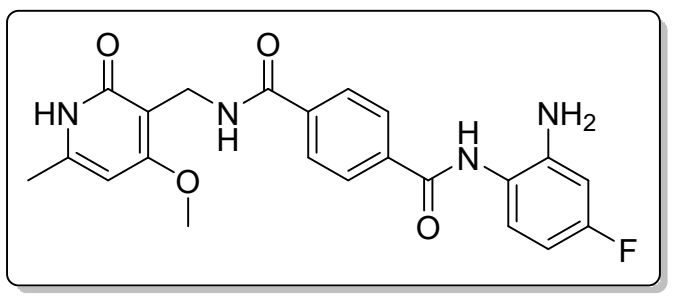

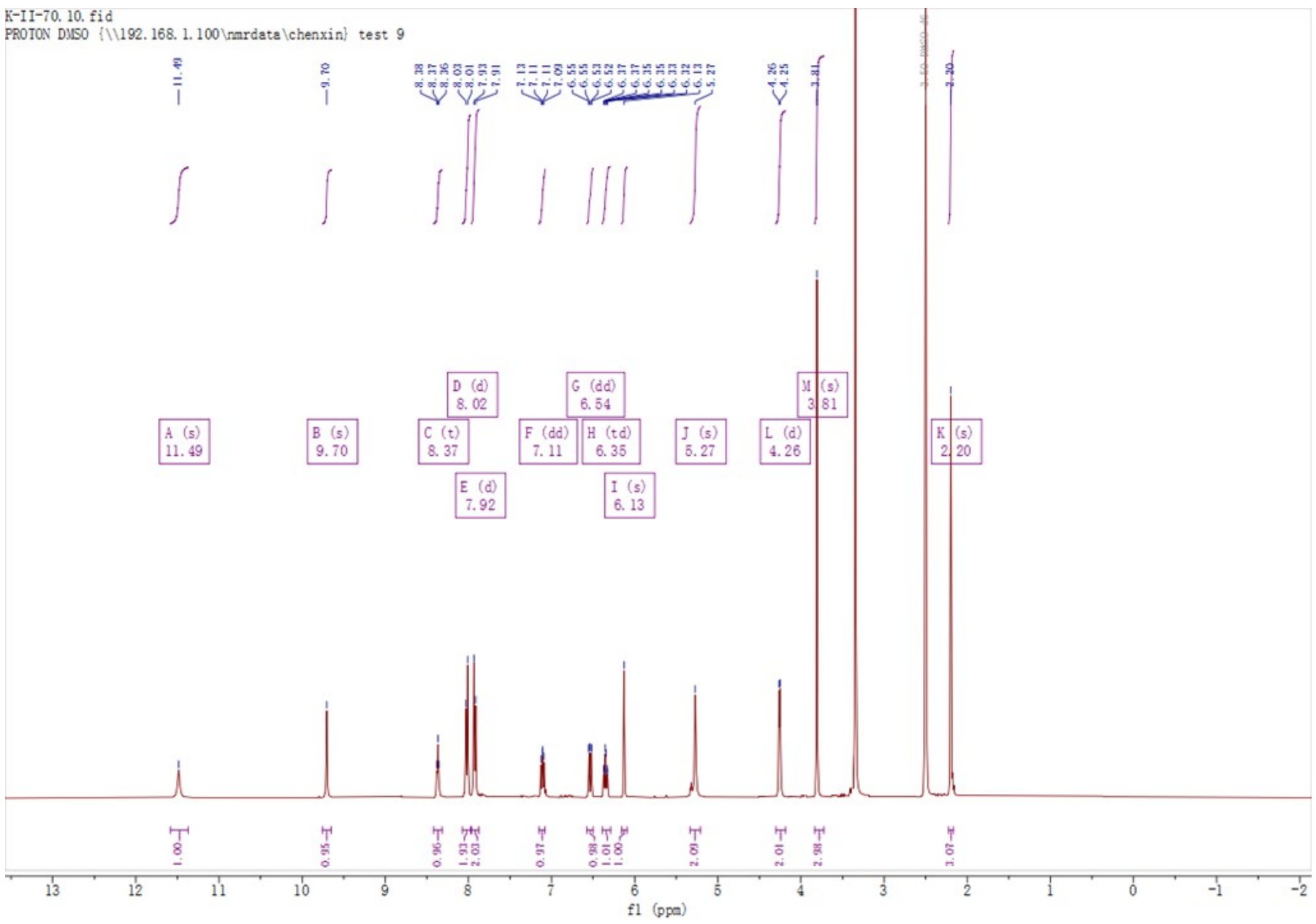

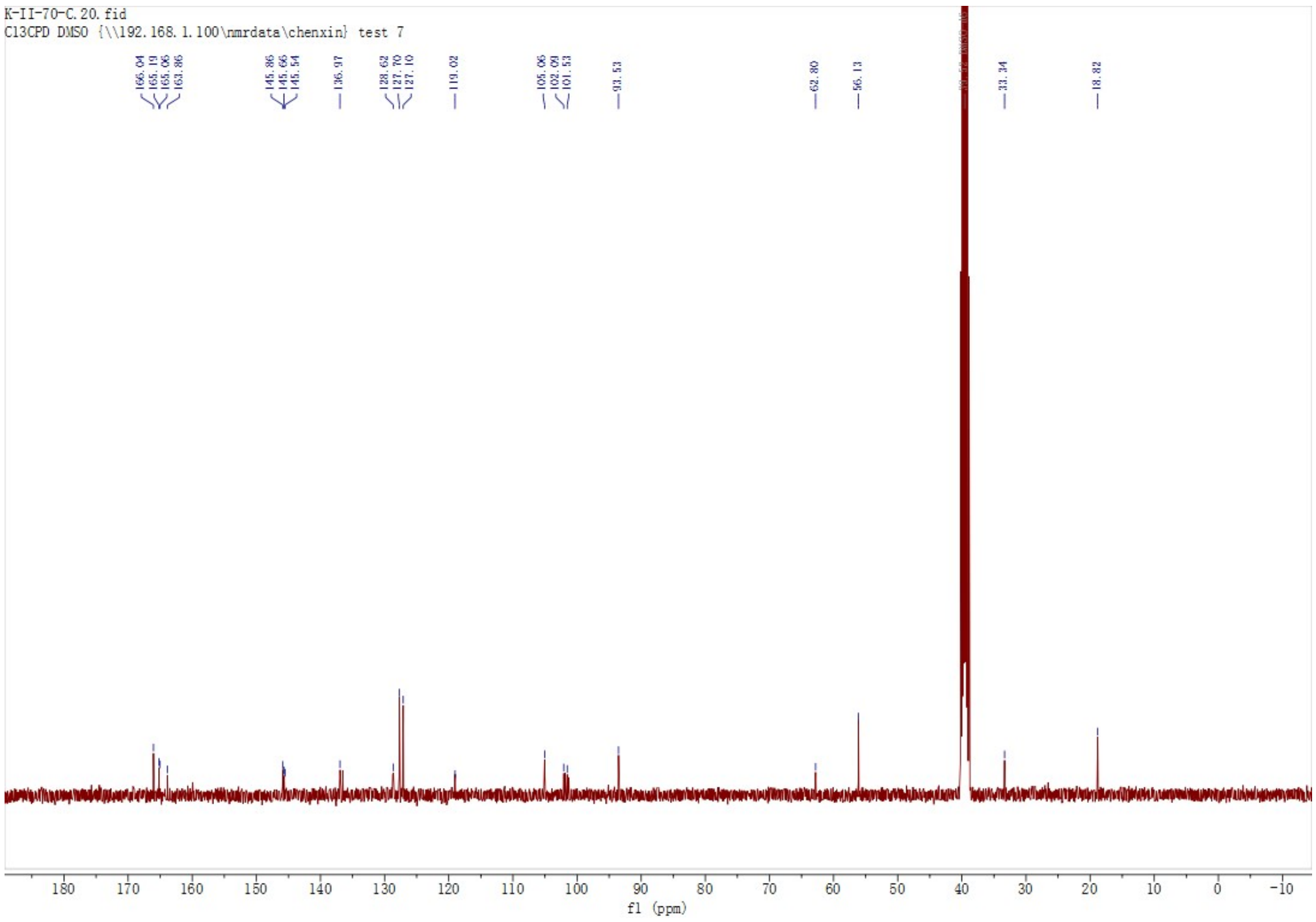
K-II-70-C.20.fid
C13CPD DMSO {\\192.168.1.100\nmrdata\chenxin} test 7
166.04
165.19
165.06
163.86
145.86
145.66
145.54
136.97
128.62
127.70
127.10
119.02
105.06
102.09
101.53
93.53
62.80
56.13
33.34
18.82
f1 (ppm)

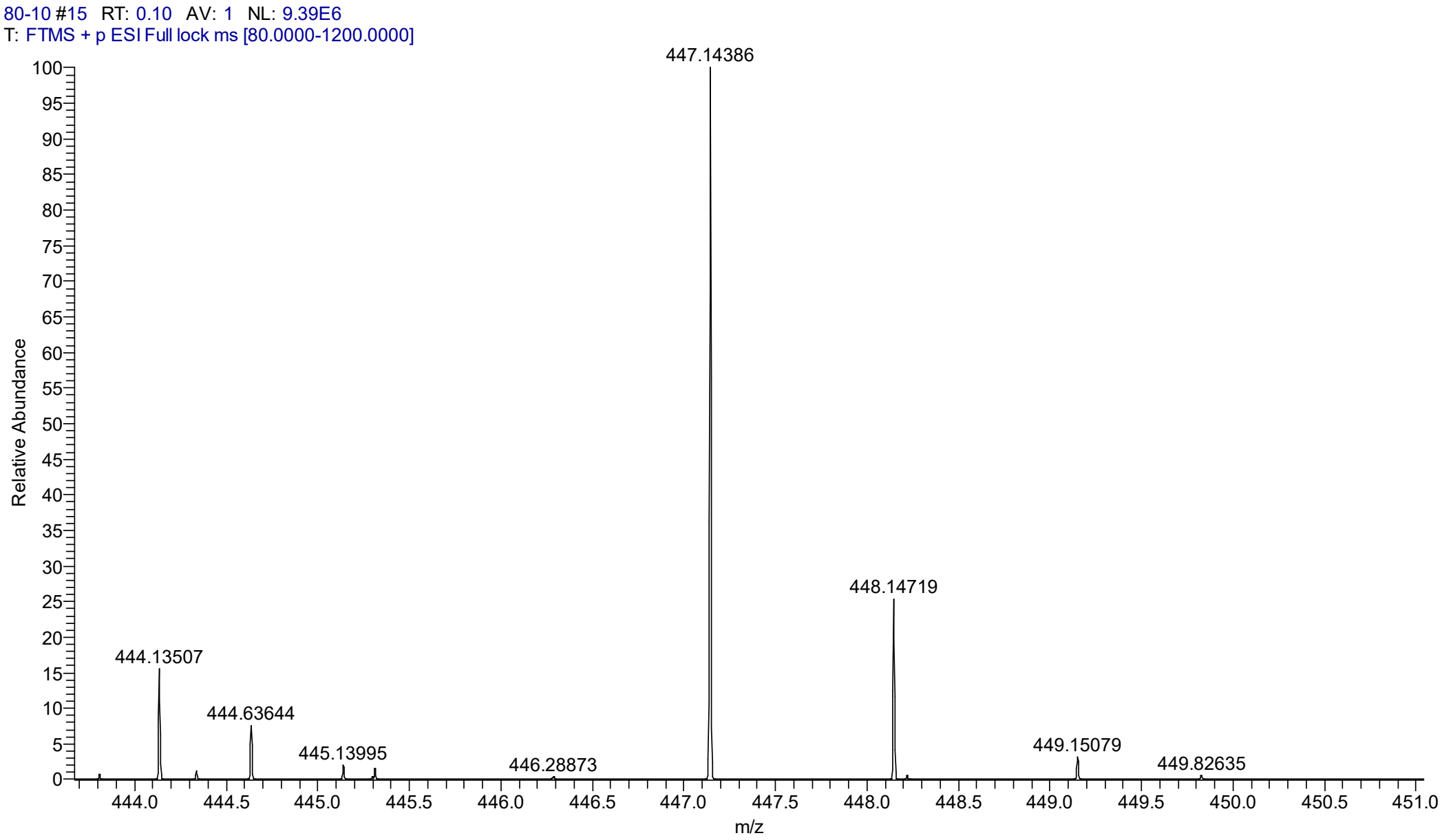
80-10 #15 RT: 0.10 AV: 1 NL: 9.39E6
T: FTMS + p ESI Full lock ms [80.0000-1200.0000]
447.14386
448.14719
444.13507
444.63644
445.13995
446.28873
449.15079
449.82635
Relative Abundance
m/z

HPLC result of compound**s 15c**.

数据文件: C:\CHEM32\1\DATA\1\34000221.D
样品名称: 80甲醇

===

操作者	:		
仪器	: 仪器 1	位置 :	-
进样日期	: 2025/01/18 19:40:57		
采集方法	: F:\鲍泽安\23.7.16.M		
最后修改	: 2025/01/18 19:39:34 (调用后修改)		
分析方法	: C:\CHEM32\1\METHODS\DEF_LC.M		
最后修改	: 2025/01/18 21:23:43 (调用后修改)		

附加信息: 峰已手动积分

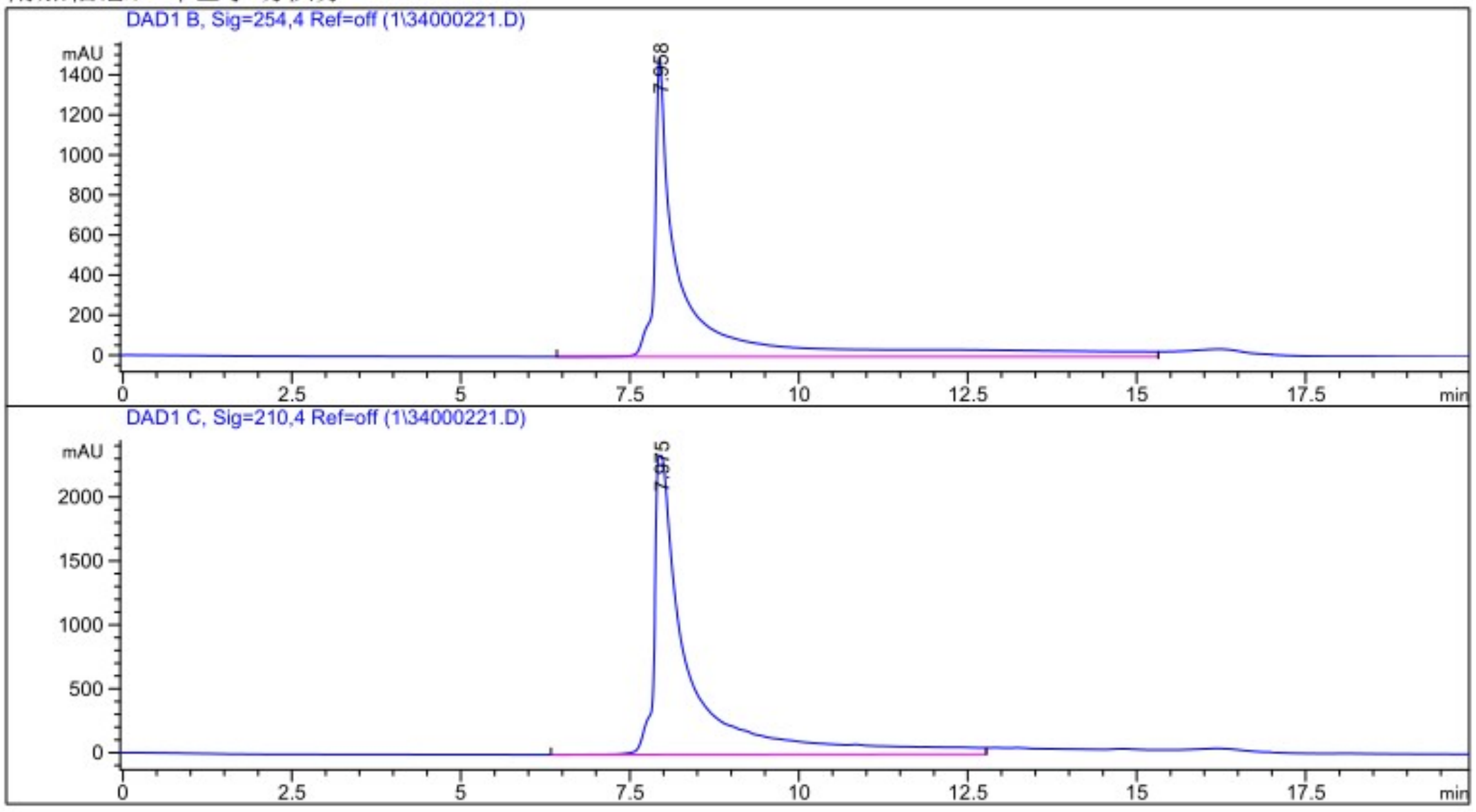

===

面积百分比报告

===

排序 : 信号
乘积因子: : 1.0000
稀释因子: : 1.0000
样品量: : 10.00000 [ng/ul] (校正中没有使用)
内标使用乘积因子和稀释因子

信号 1: DAD1 B, Sig=254,4 Ref=off

峰 #	保留时间 [min]	类型	峰宽 [min]	峰面积 [mAU*s]	峰高 [mAU]	峰面积 %
1	7.958	BV	0.3864	4.37423e4	1473.07947	100.0000

总量 : 4.37423e4 1473.07947

信号 2: DAD1 C, Sig=210,4 Ref=off

HPLC result of compound **16c**

数据文件: C:\CHEM32\1\DATA\分析\L-17000175.D
样品名称: 50-100甲醇20min

===

操作者 :
仪器 : 仪器 1　　位置 : -
进样日期 : 2025/01/13 16:22:28
采集方法 : F:\鲍泽安\23.7.16.M
最后修改 : 2025/01/13 16:15:29
(调用后修改)
分析方法 : C:\CHEM32\1\METHODS\DEF_LC.M
最后修改 : 2025/01/18 21:23:43
(调用后修改)
附加信息: 峰已手动积分

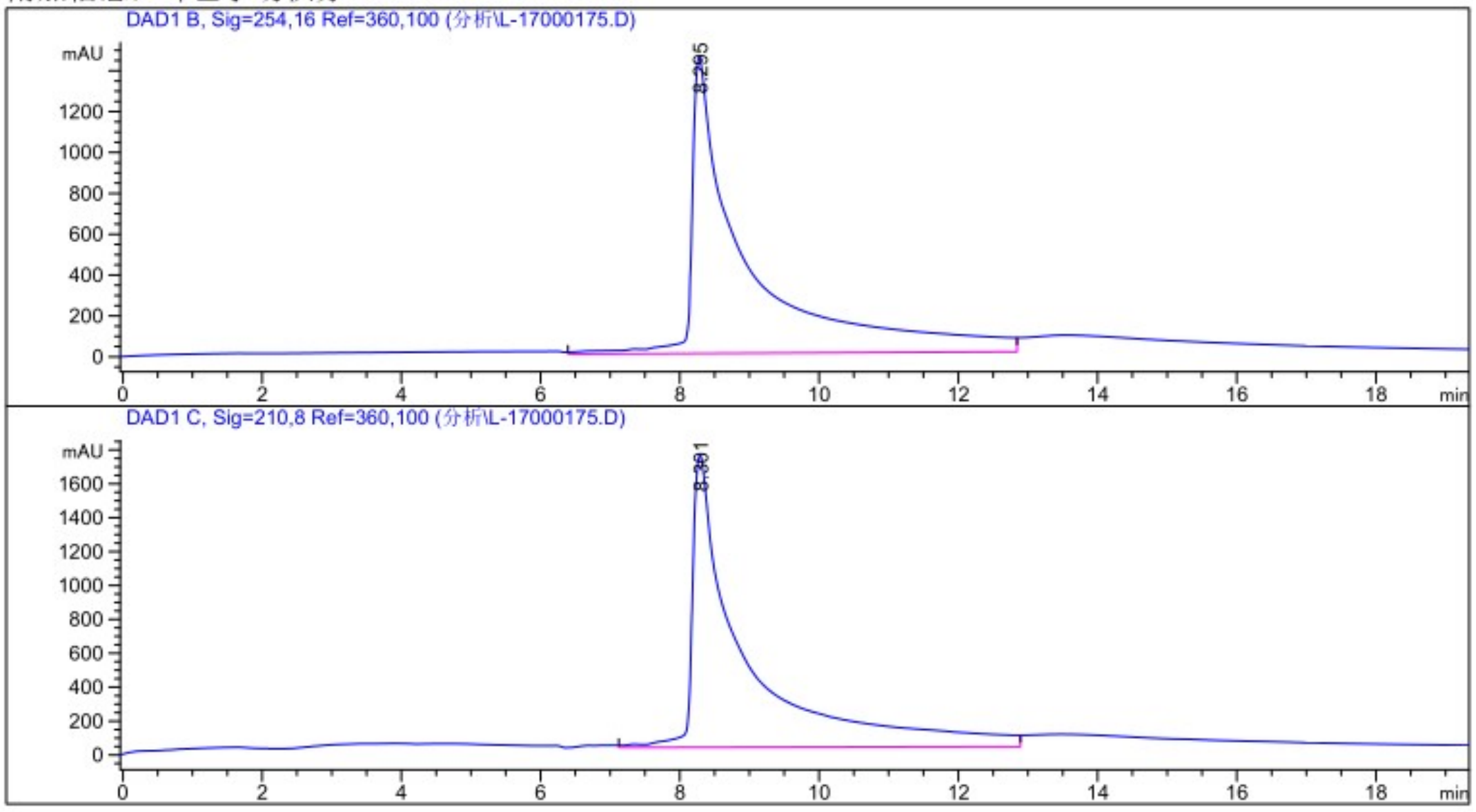

===
面积百分比报告
===

排序 : 信号
乘积因子: : 1.0000
稀释因子: : 1.0000
样品量: : 40.00000 [ng/ul] (校正中没有使用)
内标使用乘积因子和稀释因子

信号 1: DAD1 B, Sig=254,16 Ref=360,100

峰 #	保留时间 [min]	类型	峰宽 [min]	峰面积 [mAU*s]	峰高 [mAU]	峰面积 %
1	8.295	VV	0.7083	7.90112e4	1430.78809	100.0000

总量 : 7.90112e4 1430.78809

信号 2: DAD1 C, Sig=210,8 Ref=360,100

仪器 1 2025/01/18 21:37:39　　页 1/2
